# Machine learning-informed one-pot biodiesel synthesis from an optimally formulated mixed non-edible oil feedstock over magnetic sulfonated biobased catalyst

**DOI:** 10.1039/d5ra07881d

**Published:** 2025-12-18

**Authors:** Paschal Enyinnaya Ohale, Andrew Nosakhare Amenaghawon, Thomas Okpo Kimble Audu, Favour Ugbodu, Lilian Chikasi Okonkwo, Oghenerukevwe Jeffrey Oghenehwosa

**Affiliations:** a Bioresources Valorization Laboratory, Department of Chemical Engineering, Faculty of Engineering, University of Benin Benin City Edo state Nigeria pe.ohale@unizik.edu.ng andrew.ameaghawon@uniben.edu thomas.audu@uniben.edu ukevweoghenehwosa07@gmail.com favourugbodu1@gmail.com; b Department of Chemical Engineering, Faculty of Engineering, Nnamdi Azikiwe University PMB 5025 Awka Nigeria; c PECO Research Investigators Sci-Lab Anambra State Nigeria

## Abstract

The disposal of domestic and industrial nonedible oils is a major source of environmental concern. Similarly, the global energy-related environmental crises have exacerbated over the past decade. In this research, a ternary mixture of non-edible oils (MNEO) from castor oil (CO), waste cooking oil (WCO) and recovered-oil from palm oil mill effluent (RO-POME) was optimally formulated as a feed-stock for biodiesel production. MNEO formulation was achieved using a D-optimal mixture design-aided intelligent optimization. Poultry droppings (PD) was sequentially subjected to calcination, sulfonation and magnetization to yield a reusable heterogeneous catalyst. One-pot transesterification was modeled using explainable machine learning algorithms including support vector regression (SVR), artificial neural network (ANN), and eXtreme Gradient Boosting (XGB) followed by Manta ray foraging optimization (MRFO). The optimally formulated MNEO comprised 21.31% WCO, 18.45% RO-POME, and 60.24% CO, with improved properties compared to individual oils. Fatty acid profiling of MNEO revealed it contained 29.96% (saturated), and 64.7% (unsaturated) fatty acids. Characterization results revealed the potentials of Fe_3_O_4_@CPD–SO_4_ in facilitating MNEO transesterification reaction. Comparative modeling demonstrated satisfactory applications of ANN, SVR and XGB, while error indices established XGB as the most superior model in capturing the complex nonlinear dynamics of the system. Feature ranking established methanol–oil molar ratio as the most influential parameter predicting biodiesel yield, underscoring the important role of methanol in biodiesel synthesis. Furthermore, optimum reaction temperature, catalyst dosage, methanol–oil-ratio and reaction time of 50 °C, 3.01 wt%, 30.0, and 2.4 h, obtained from XGB-MRFO resulted in a yield of 99.68% which was experimentally validated to be 98.16%. It is concluded that MNEO and poultry droppings can be successfully employed for sustainable biodiesel synthesis.

## Abstract 2

The disposal of domestic and industrial nonedible oils is a major source of environmental concern.

## Graphical abstract

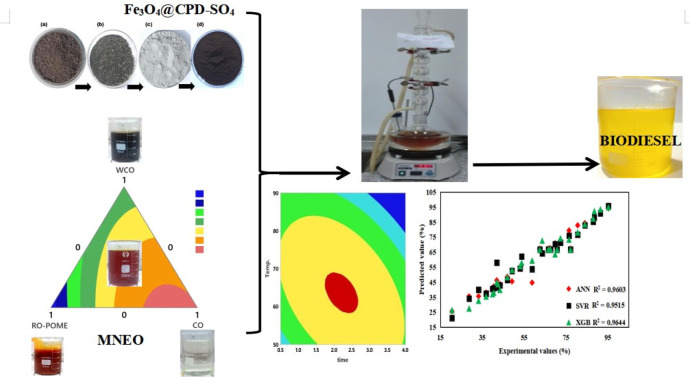

## 1. Introduction

The global community is currently apprehensive about energy usage from fossil sources, owing to their non-renewable nature and associated environmental consequences.^1^ Promoting natural, renewable, sustainable, and cost-effective energy is vital because of the negative environmental impacts of the ongoing use of conventional energy resources (coal and petroleum) cannot be disregarded, especially as concerns about climate change continue to grow.^2^ Given that biodiesel is a sustainable energy source made from natural resources and shares several qualities with conventional diesel fuel, it can be adopted as a substitute for fossil based petrodiesel in internal combustion engines.^3^

According to literature, one of the major factors impeding the sustainable production of biodiesel is the cost of oil feedstock which accounts for over 80% of the overall process cost.^4^ Biodiesel was predominantly produced from edible oil feedstock some of which include soybean oil,^5^ palm oil,^6^ sunflower oil.^7^ However, the utilization of edible oils have raised concerns on feedstock availability, cost, and food supply shortages, motivating researchers to seek for alternative nonedible sources.^8^ Consequently, nonedible oils including castor oil,^9^ waste cooking oil,^10^ lipids,^11^ waste animal fats^12^ are now preferred because they are less expensive feedstocks for cost effective biodiesel synthesis.

Despite these process cost reduction advantages of employing nonedible oils, the performance of biodiesel obtained from these single feedstocks, with respect to oxidative stability and cold flow qualities, requires significant improvement prior to use.^13^ Several strategies have been suggested out to address these limitations, and top on the list is the blending of oil feedstocks. Blending nonedible oil feedstocks is being proposed as an economical method to produce biodiesel with enhanced qualities.^14^ In addition, blending of oil feedstocks is a mutually beneficial approach where the favorable features associated with each oil is optimally exploited to form a blend which exhibits superior attributes relative to each of the constituents.^15^ Even though there have been a number of previous endeavors to investigate the feasibility of exploiting biodiesel feedstocks containing a variety of oil species, the full potential of this strategy has been constrained by trial-and-error approaches.^16^ The process of trial-and-error possesses identical drawbacks to the one-factor-at-a-time experimental procedure. It cannot ensure the expected optimum and it limits the researcher from investigating all of the factor distributions of every parameter.^14,17^ In implementing a mixture experimental design as opposed to the trial-and-error method, these limitations can be avoided.

Catalysts are essential in the transesterification reaction as they enhance the reaction rate and reduce the activation energy required for the reaction, hence accelerating and optimizing the process.^18^ Homogeneous catalysts including sulfuric acid, potassium hydroxide, and sodium hydroxide have been useful in biodiesel production.^19^ Although these catalysts have been recorded to have the advantages of facilitating mild reaction conditions, improved reaction rates, and high biodiesel yields, they are also associated with separation difficulties which significantly increases the process costs.^20^ As a result, researchers have focused attention on developing heterogeneous catalysts that facilitate biodiesel production, enhance reusability to lower process costs, and provide environmental advantages by minimizing waste.^5^ The catalytic performance of some catalysts synthesized from biobased calcium rich precursors reported in literature have been significantly satisfactory, with respect to biodiesel yield.^21–23^ For instance, Goli *et al.*^21^ investigated the transesterification of soybean oil using CaO derived from chicken egg shells, and obtained an optimum yield of 92.32% at a reaction time of 3 h. Similarly, Gaide *et al.*^22^ employed snail shell based CaO catalyst to obtain a biodiesel yield of 98.15% at a reaction time of 8 h using rapeseed oil, even as Maneerung *et al.*^23^ obtained 90% biodiesel after 5 h using CaO catalyst from chicken manure and WCO feedstock. Despite the recorded satisfactory yields, these catalyst species suffer some peculiar drawbacks which include long reaction time, accelerated leaching of active sites during reuse and less catalytic activity compared to homogeneous catalyst.^17^ In order to address these issues, researchers have successfully incorporated sulfonation,^24–26^ and magnetization^27,28^ procedures which improved catalytic activity. The use of magnetite (Fe_3_O_4_) has the dual benefit of its magnetic capability which facilitates effective magnetic separation with reduced leaching as well as offering special catalytic function. Biobased poultry droppings (PD) primarily constitute fibrous manure rich in carbon, calcium, potassium, phosphorous and other micro-nutrients (nitrogen, magnesium, zinc, *etc.*). The satisfactory performance of valorized poultry droppings in the catalysis of transesterification reaction has been documented.^23,29^ In this work, poultry droppings (PD) was selected as a catalyst precursor because of its rich content of alkali (potassium) and alkaline earth metals (calcium, magnesium) which is a major advantage over previously reported biomass wastes. In addition to this, PD is also cheaply available all year round.

Transesterification reaction of fatty acids is a multi-variable process whose efficiency depends majorly on temperature, molar ratio of methanol to oil, catalyst dosage, and reaction time.^30^ One-factor-at-a-time (OFAT) experimental technique has extensively been applied to investigate the impact of these variables on transesterification reaction.^31,32^ However, the OFAT technique is time consuming, capital intensive and does not guarantee the search for desired optimum. Consequently, statistical techniques such as response surface methodology (RSM) have been developed to overcome these limitations. However, reports have demonstrated that RSM encounters difficulties with intricate, non-linear interactions and necessitates meticulous design of experiments (DOE) for process optimization, which may be less effective and costly.^33^ Recently, many research efforts have been dedicated to investigating transesterification reaction using machine learning (ML) models. These models include kernel-based support vector regression (SVR),^34^ tree-based extreme gradient boosting (XGB),^35^ and neural network-based artificial neural network.^36^ Employing machine learning algorithms in transesterification reaction offers a powerful approach for data acquisition, interpretation and prediction of methanolysis process dynamics *via* pattern recognition which is superior to the OFAT and RSM procedures.^34,35^ A generic overview of chemical systems exploring a comparative analysis of machine learning algorithms have yielded conflicting conclusions regarding the superiority of any modeling technique.^37^ While executing ML technologies for modeling of biodiesel production, evaluating model uncertainty and designation relative priority of input features are two aspects that are frequently ignored. These attributes, however, can offer insightful information about the model. In order to overcome this, SHapley Additive Explanation (SHAP) is preferred because it assigns the model's uncertainty to distinct points of variation that stem from input features, providing benefits superior to analysis of variance (ANOVA).^17^

Despite the fact that many studies have recorded more than 90% conversion of mixed non-edible oils to biodiesel,^38–40^ the scientific purpose of blending these feedstock remains largely unreported. For instance, in most of the reported literature, the major reasons for blending non-edible oils were to increase biodiesel yield, and to circumvent the complexities of feedstock cultivation. However, a more scientific justification for feedstock blending is to optimize key feedstock properties including acid values (AV), iodine value (IV), and density (*ρ*) of the blended product. A typical exemplification is the deployment of high AV laden feedstock such as recovered oil (RO) from palm oil mill effluent (POME, RO-POME) in biodiesel production which has never been reported in literature. Consequently, it is highly desirable to optimize the quality parameters of low-grade RO-POME feedstock though blending. In order to achieve this, mixture design technique is used, as opposed to trial and error approach.^14^ To the best of our knowledge, the implementation of the mixture design approach in optimal formulation of feedstock blends remains largely unreported. Also, it is important to highlight that even though literature review demonstrated the potentials of poultry droppings in forming a catalyst basis for transesterification reaction, its application in either pure or modified forms remains significantly undocumented. There is no published research on the application of sulfonated magnetic poultry droppings as a catalyst for biodiesel production.

Information deduced from literature showcased the obvious distinct topological differences between the considered machine learning algorithms. Given these distinct network-topology (neural network, kernel and tree) of ML algorithms, it becomes highly desirable to comparatively assess the performance of SVR, ANN, and XGB in transesterification reaction of the present system. To the best of our knowledge, no report has been put out on comparative assessment of ML algorithms in transesterification of blended oil feedstock based on their topological differences. Thus, the current research focused on the application of an optimally formulated ternary oil as a feedstock for methanolysis reaction in the presence of magnetic bi-functional catalyst from poultry droppings. Specifically, the objectives of the work include the optimal formulation of ternary mixed non-edible oil (MNEO) feedstock comprising WCO, RO-POME, and CO using mixture design. Also, the preparation and characterization of bi-functional heterogeneous catalyst from poultry droppings were carried out. The work carefully considered the comparative modeling and optimization of one-pot transesterification reaction using ML algorithms (SVR, ANN, and XGB), sensitivity analysis and optimization with SHAP and Manta ray foraging optimization (MRFO), respectively. Catalyst reusability and characterization of synthesized biodiesel and spent catalyst were extensively investigated.

## 2. Materials and methods

### 2.1. Procurement and preparation of raw materials

Waste cooking oil was collected from food outlets and fast food restaurants in Awka, Anambra State Capital, Nigeria. The collected WCO was allowed to settle for 7 days at ambient temperature and pressure before filtration through a screen size of 100 nm to eliminate any food residues and inorganic remnants, and then dehydrated at 110 °C for 12 h. Palm oil mill effluent (POME) was obtained from a palm oil processing factory at Amaenyi, Anambra State, Nigeria. The collected POME sample was sieved through a screen size of < 50 µm, and allowed to stand for 10 days under room temperature and pressure, after which the sample was screened using 100 nm Whatman filter paper and dehydrated at 110 °C using a laboratory oven. Castor seeds were locally sourced from farmers in Amansea, Anambra State, Nigeria. The seeds were sun-dried at an average temperature of 33 °C to constant weight, and then dehusked before grinding to particle size of < 150 microns. The wet castor seed cake was mechanically expressed using a screw conveyor to obtain the castor oil. The extracted oil was dehydrated using a Memmert oven at a temperature of 105 °C. The moisture-free oil was labeled and stored for further processing.

Fresh poultry droppings were sourced from Ochendo poultry farm, Anambra State, Nigeria. The poultry droppings were magnetically separated to remove all metal substances, after which they were was sun-dried and then pulverized using a mechanical crusher. The pulverized samples were preserved in a cold vessel at a temperature of 4 °C until further use.

Analytical grade reagents of sulfuric acid (assay > 98%), and sodium sulfate (Na_2_SO_4_) which were deployed in the catalyst activation and biodiesel refining steps, respectively, were supplied by Parchem Limited, New Rochelle, New York, USA. Also, ferric chloride hexahydrate (FeCl_3_·6H_2_O), ferrous chloride tetrahydrate (FeCl_2_·4H_2_O)and ammonium hydroxide (NH_4_OH) which were used for the magnetisation process were supplied by Molychem chemical industry, Badlapur, Dist. Thane 421 503, India.

### 2.2. Methods

#### 2.2.1. Catalyst preparation and characterization

The catalyst was prepared by calcining the poultry droppings at 850 °C at a rate of 10 °C min^−1^ in a muffle furnace for 2 h.^41,42^ After this, the sample was allowed to cool to room temperature to obtain calcined poultry droppings (CPD). The CPD was sulfonated by mixing 1 g of CPD with 6 mL of 5 M H_2_SO_4_ at a temperature of 90 °C for 6 h^41,43–45^ to introduce Brønsted acid sites. The sulfonated CPD (CPD–SO_4_) was filtered and dried in an oven at 105 °C for 30 min, after which the dry sample was washed with excess distilled water until neutral pH.

The synthesis of magnetic sulfonated CPD catalyst (Fe_3_O_4_@CPD–SO_4_) was executed in two steps. Firstly, Fe_3_O_4_ was prepared by co-precipitation. For this step, 59.64 g and 162.198 g of FeCl_2_·4H_2_O and FeCl_3_·6H_2_O, respectively, were dissolved in 300 mL of aquadest under constant stirring until complete dissolution. Subsequently, 0.5 M NH_4_OH solution was added slowly in a drop wise manner under constant stirring conditions (500 rpm) until a pH of 11 was attained. This process was maintained at 60 °C for 20 min to obtain a Fe_3_O_4_ solution. Furthermore, the wet impregnation method was employed to prepare the final catalyst by adding 100 g of CPD–SO_4_ in 300 mL of Fe_3_O_4_ solution, and vigorously stirring at 500 rpm for 5 h. The obtained precipitate was washed with excess water, and oven dried at 60 °C to obtain Fe_3_O_4_@CPD–SO_4_.^28,46,47^ The magnetic catalyst was stored in an airtight container for further characterization and use.

**Fig. 1:**
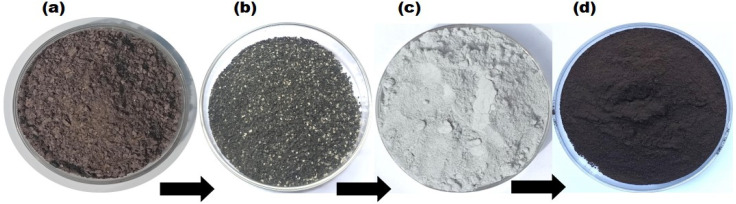
Catalyst species preparation steps showing (a) raw poultry droppings, (b) calcined poultry droppings (CPD), (c) sulfonated calcined poultry droppings (CPD–SO_4_), (d) magnetic–sulfonated/calcined poultry droppings (Fe_3_O_4_@CPD–SO_4_).

Fig. 1 depicts the sequential transformation of poultry droppings into Fe_3_O_4_@CPD–SO_4_. While a typical sample of poultry droppings appear brownish-black as a result of microbial induced fermentation (Fig. 1(a)), the calcined (Fig. 1(b)) and sulfonated samples (Fig. 1(c)) appear black and pale-grey, respectively. The final magnetic catalyst in Fig. 1(d) were stored in an airtight container for further use.

The physicochemical characterization of FPD, CPD, CPD–SO_4_, and Fe_3_O_4_@CPD–SO_4_ catalyst was carried out using instrumental analyses. The surface chemical functional groups in all species were identified using FTIR-Thermo Nicolet Nexus (Model 470/670/870) at infrared absorption bands within the range of 400 to 4000 cm^−1^. The morphological topographic features and associated elemental composition of FPD and Fe_3_O_4_@CPD–SO_4_ was investigated by scanning electron microscope (SEM-Model Zeiss Evo MA-17 EDX/WDS microscopy). Crystallographic features if FPD and Fe_3_O_4_@CPD–SO_4_ were obtained by the reflection scan using XRD-Philips XPERT X-ray diffraction unit. The unit was operated at 40 kV and 609 mA. All readings were performed at 2*θ* values within 10 and 80°. The thermal stability and thermal decomposition studies of FPD, CPD, and Fe_3_O_4_@CPD–SO_4_ were assessed using thermo-gravimetric analysis TGA-Mettler Toledo TGA/SDTG 851. The specific surface area of Fe_3_O_4_@CPD–SO_4_ was explored using Brunauer–Emmett–Teller (BET) technique, while the pore volume was examined using Barrett–Joyner–Halenda (BJH) technique and at *p*/*p*^o^ = 0.99.

#### 2.2.2. Pretreatment and formulation of non-edible oil blend

Oil recovery from palm oil mill effluent was performed according to the procedure recommended by other researchers with some modifications.^48,49^ The filtered and dehydrated POME sample was mixed with hot water (>80 °C) in a ratio of 1 : 3 (wt of POME: vol. of water), and heated to 100 °C in a water bath. During the heating process, the mixture was slowly stirred to ensure a clear separation of oil at the top, with sludge and water at the bottom. The hot mixture was put into a separating funnel for oil recovery and water removal.

The formulation of the oil blend from recovered oil from POME, castor oil and waste cooking oil was done according to a D-optimal mixture design implemented in the Python package PyDoE2.^17^ This design is preferred by researchers in formulation development due to its benefits, which comprise reduced fluctuation in model parameter estimates, complete representation of the design domain, reduced experimental runs, and enhanced model stability.^50^Table 1 displays each component of the blend together with their respective value ranges, whereas eqn (1) and (2) specify the relative amounts of these components.

1 WCO + RO − POME + CO = 100%

2 0 ≤ WCO, RO − POME, CO ≤ 100%

**Table 1:** Value distribution of MNEO constituents for ternary feedstock composition

Variable	Variable levels	
	Low	High
WCO (%)	0	100
RO-POME (%)	0	100
CO (%)	0	100

For the actual formulation, each volume of oil sample was dispensed into a conical flask to form a blend according to the design outlined in Table 1, and the oil blend was stirred vigorously, using a magnetic hot plate stirrer. The process was allowed to continue for 1 h, after which the homogeneous oil mixture was collected and characterized for acid value, density, and iodine value which served as response variables.

#### 2.2.3. Biodiesel production studies

The biodiesel reaction was carried out in a three-neck flat bottom flask fitted with Dimroth condenser. A slightly modified method used by Dharmalingam^51^ was adopted for this procedure. Firstly, predetermined amounts of methanol and catalyst were measured and mixed at 60 °C for 40 min. in the three-neck flask. Also, specified amount of the oil blend which had been preheated at 50 °C for 1 h was added to the three-neck flask containing methanol-catalyst mixture to maintain a desired methanol-to-oil molar ratio. After adding the formulated oil blend, the temperature of the setup was adjusted to the desired transesterification reaction temperature and left to continue at a stirring speed of 400 rpm until the end of the process. At the end of the reaction, the reactants were discharged into a separating funnel where the catalyst and other products was recovered *via* decantation after 24 h. The crude biodiesel was refined by gently washing multiple times with warm water. The washed biodiesel was dried over anhydrous Na_2_SO_4_, while the recovered catalyst was washed with methanol and dried at 70 °C for 2 h. The percentage yield of biodiesel was estimated using the eqn (3).^38,51,52^3
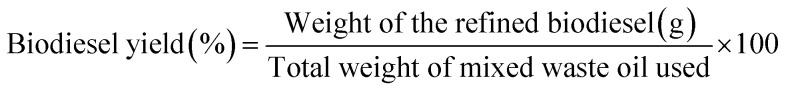


#### 2.2.4. Characterization of non-edible oil mixture and biodiesel product

Standardized experiments were performed to characterize the mixed non-edible oil and biodiesel product. Specifically, MNEO and biodiesel product were characterized to obtain the acid value, free fatty acid (FFA), viscosity, saponification value, iodine value, and density. Furthermore, key fuel properties including cloud point, pour point, moisture content, cetane number, higher heating value and methyl ester were used to ascertain the qualities of the biodiesel product. All tests were conducted using ASTM D6751 standards.^53^

### 2.3. Experimental design and machine learning modeling

The biodiesel synthesis procedures were designed utilizing a central composite design (CCD) featuring four factors related to the input set of parameters: reaction temperatures, catalyst dosage, reaction time, and the molar ratio of methanol-to-MNEO. The selection of the CCD stemmed from the benefits it offers, which include reliable stability of the design model, reduced sampling errors due to a five-level spread of design data, the ability to conform to quadratic effect, statistical accuracy in computing parameter values, and satisfactory interpolation attributes.^33^ The design framework was created with the Python package PyDoE2, employing the inputs presented in Table 2, which were established from initial analyses and references in literature.^17^ The data produced by the CCD was utilized to develop machine learning models for the production of biodiesel. All studies conducted during the machine learning implementation process were performed in Python (version 3.7.5) utilizing Jupyter as a unified development framework. For the numerical assessment, Numpy software version 1.17.0 was used, while OriginLab (2025b) was employed for data visualization and graph creation. Furthermore, a heatmap of Pearson correlation was used to evaluate and visualize the impact of each feature on the measured targets, and this was created using OriginLab (2025b). The heatmap makes it easier to rapidly determine whether variables are substantially positive, negatively correlated, or weakly connected by using color intensity to reflect the correlation degree (which ranges from −1 to 1). Three machine learning algorithms including SVR, ANN, and XGB were used to predict the transesterification reaction of MNEO using Scikit-learn version 0.23.2, TensorFlow version 2.4.0, and XGB version 1.2.3, respectively.

**Table 2:** Factor levels for transesterification reaction

Factor	Factor levels				
	Level 1	Level 2	Level 3	Level 4	Level 5
Reaction temperature (^o^C)	50	60	70	80	90
Catalyst dosage (wt%)	1	2	3	4	5
Methanol-to-oil molar ratio	6	12	18	24	30
Reaction time (h)	0.5	1.38	2.25	3.13	4.0

Given their complementing algorithmic flexibility and demonstrated efficacy in process modelling incorporating nonlinear and multivariate systems, ANN, SVR, and XGB were chosen for the present study.^17^ For instance, SVR incorporates a concept identified as structural uncertainty minimization, which enables it to obtain satisfactory generalization ability and minimize over-fitting, rendering it particularly appropriate for small datasets.^54^ Also, XGB, a gradient-driven tree network, has shown remarkable performance in systems that are marked by unpredictable and complex information due to its regularization capabilities and scalability.^55^ Lastly, ANN excels in identifying complicated patterns and has become widely applied in modelling chemical engineering systems containing both large and small amounts of data.^56,57^ The employment of these three algorithms ensures an accurate evaluation across essentially unique computational frameworks, hence boosting the robustness of prediction insights and guaranteeing a thorough model selection for optimizing biodiesel yield.

While it is generally believed that machine learning (ML) algorithms require large amount of data for modeling, several publications have demonstrated that, in fact, provided that a dataset is statistically well distributed across a design space, it will be sufficient to execute ML modeling. Some experimental designs that have been used to achieve such statistical data distribution for ML algorithms in biodiesel synthesis include Box–Behnken design,^58,59^ and central composite design.^60–62^ Specific data points assigned for training and testing have been highlighted in Table S2 (SI). For hyperparameter tuning, the 30 biodiesel experimental dataset was split into 80% (24 data points) 20% (6 data points) for training and testing purposes respectively, and then used for a five-fold cross-validation procedure. The entire set of training data was used for cross-validation, and the verification dataset, which hadn't been given to the models for their training phase, was used for testing. This was performed in order to reduce the possibility of overfitting and improve the model's capacity to generalize adequately to new data, especially for the case of ANN. Further hyperparameter tuning was done using Bayesian optimization.

#### 2.3.1. Artificial neural network

A neural network with a feed-forward structure was used in the present investigation. A group of neurones that make up the ANN's hidden layer was employed to evaluate the networks prediction (*y*) from the supplied feature data (*x*). The neurone uses appropriate biases (*b*) and weights (*w*_*i*_) to estimate the feature characteristics (*x*_*i*_) by computing linear combinations (*z*) using eqn (4). The selection of the activation algorithm is an essential step in the ANN modelling process, given that it largely controls how inputs are mapped to outputs. In order to identify the best choice, a number of activation algorithms were evaluated, comprising exponential, scaled exponential linear unit (SELU), rectified linear unit (ReLU), and leaky rectified linear unit (Leaky ReLU).^17^4
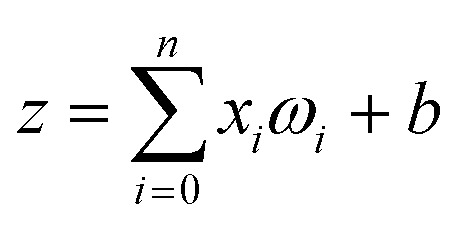


The predictive network was developed using an iteration method for the ANN technique, which uses the root mean square error (RMSE) as an indicator (eqn (5)) to modify the prediction weights in order to minimize the model loss 
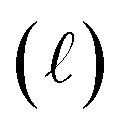
. This is accomplished by the use of a gradient-descent algorithm, which scales weights employing the pattern recognition rate (*λ*) and adjusts them depending on overall loss of model information (eqn (6)). To optimize the ANN hyperparameters, grid-based cross validation (GridSearchCV) was used.5
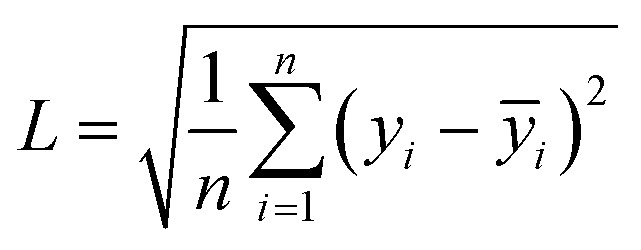
6
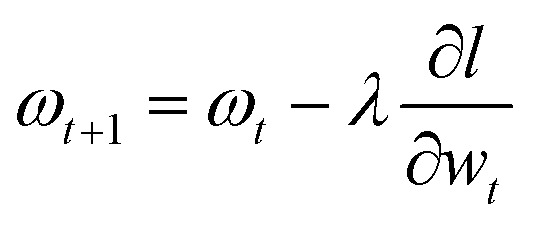
where experimental and predicted data were denoted as *y*_*i*_ and *ȳ*_*i*_, respectively.

#### 2.3.2. Support vector regression

Support vector regression (SVR) is a customized functional unit of support vector machine (SVM), tailored for the purpose of regression analysis. Support vector machine is equipped with a sophisticated, controlled learning kernel-based algorithm machine learning framework. They are especially helpful in processing high-dimensional data, such as those found in bioenergy research, given that they could possibly be used to represent complex situations.^63^ The SVR seeks to create the optimal hyperplane for discriminating between various data source categories. Denoting a vector of targets as *y* = *R*^*n*^, and input features as *x* = *x*_*i*_, ∈*R*^P^; *i*, …, *n*, a model regression for SVR is generated in eqn (7).

7 *F*(*x*) = *ω*^T^*ϕ*(*x*) + *b*

The weights (*ω*) and biases (*b*) are determined by subjecting eqn (8) to minimization.8
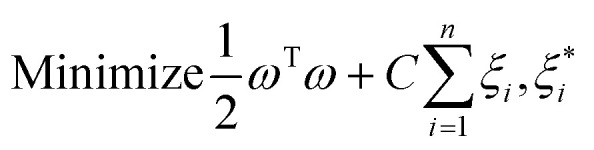


Subject to:9
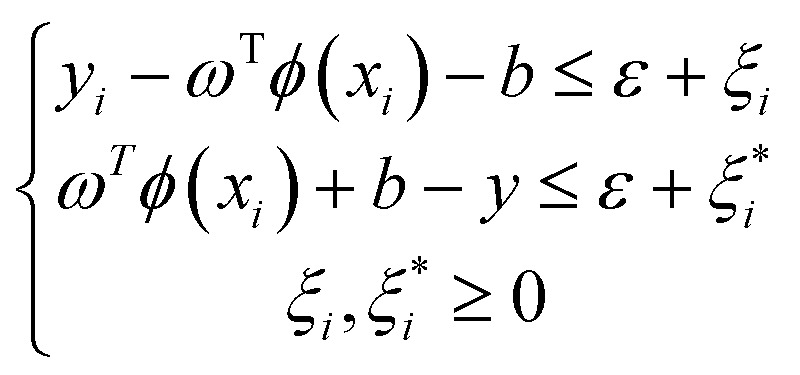
*ξ* and *C* are error and penalty terms, respectively, associated with the SVR hyper parameters. Consequently, the SVR predictions are given in eqn (10).10
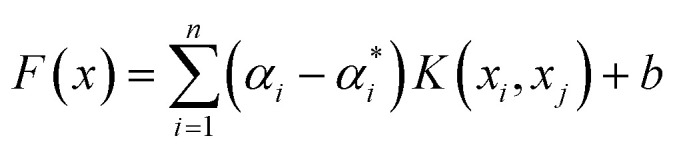
where *α*_*i*_ and 
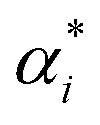
 are Lagrange multipliers and, *K* is the kernel. To choose the best, sigmoid, linear, polynomial, and radial basis function (RBF) kernels were evaluated.

#### 2.3.3. Extreme gradient boosting

Another cutting-edge machine learning approach that combines decision trees and gradient-boosting techniques is called XGB. Its effectiveness comes from using progressive learning to merge many weak learners into an efficient learner. Through the use of autonomous parallel computing, XGB improves training precision and computational effectiveness. Eqn (11) shows the XGB prognosis function for a given time period (*t*). Regularization is also used by XGB to reduce overfitting of data.11
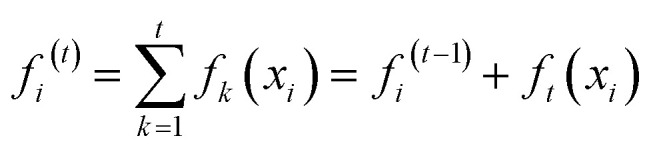
where input data, step *t* pattern learner, step *t* data prediction, and step *t* − 1 data prediction are *x*_*i*_, *f*_*t*_(*x*_*i*_), *f*^(*t*)^_*i*_, and *f*^(*t*−1)^_*i*_, respectively.

#### 2.3.4. ML model performance assessment

Four significant metrics were used to assess the ML models' functionality following hyperparameter optimization. These model appraisal techniques are useful in ranking the performance of each ML algorithm in capturing the convoluted non-linear nature of the present system. The specific error indices used in this study include coefficient of determination (*R*^2^), mean square error (MSE), root mean square error (RMSE), and Akaike's information criterion (AIC). Mathematical implications of these models are outlined in eqn (12)–(15).12
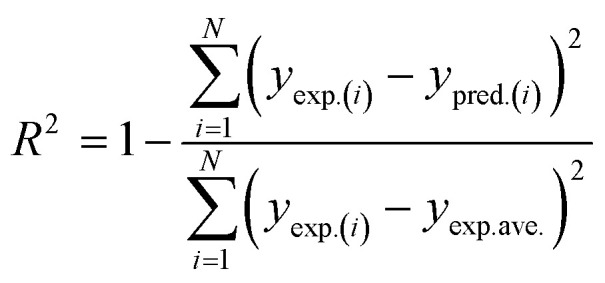
13
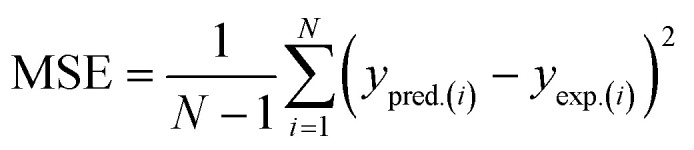
14
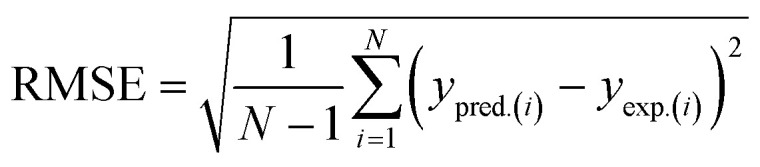
15
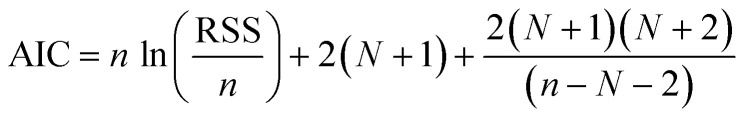


#### 2.3.5. SHAP feature evaluation

Developing an efficient machine learning model logically leads to assessing the impact of input parameters on the target variable. A relationship between characteristics and results is visualized and measured using SHapley Additive exPlanations (SHAP).^64,65^ SHAP is a flexible method for analyzing specific forecasts as well as global evaluations. SHAP uses Shapley values, which are optimum integrals, to determine the significance of features based on game theory. SHAP was selected for feature evaluation because it offers additional case-specific, in-depth interpretations that indicate the tendency and size of each feature's relevance to a prediction, compared to the Pareto effect which only highlights the most influential characteristics. SHAP technique guarantees equitable distribution of contributions over feature values, all of which is crucial for fostering model trust. Here, the significance of the input attributes influencing each target is determined using the Python SHAP module. As demonstrated in eqn (16), SHAP measures the improvement in overall effectiveness when a parameter is indicated, thereby quantifying the impact of each feature.16
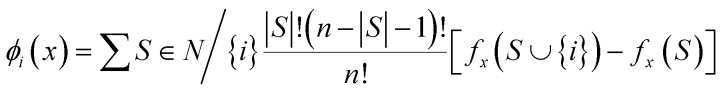
where *F* is the set of all features, *S* is a subset of features excluding *i*, and *f*_S(*X*_s_)_ is the model prediction when only features in *S* are considered.

### 2.4. Intelligent optimization

Intelligent optimization was carried out using Manta ray foraging optimization (MRFO) algorithm. MRFO is conceptualized from mimicking three foraging techniques exhibited in Manta Rays including chain, somersault, and cyclone foraging.^66^ In MRFO, manta rays are arranged from its head to its tail to create the foraging chain. The next agent, represented by *x*_1_(*t*), is thereafter modified depending on the best possible position and its neighboring solution at iteration (*t*). After that, an agent can move closer to the nutritional supply. The following is a representation of this procedure:17
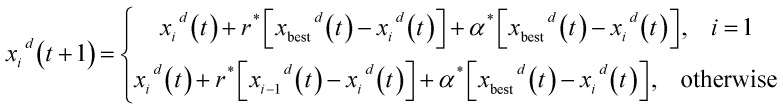
Here, *r*∈[0,1] stands for a basic vector, and *x*_best_^*d*^(*t*) is the top-performing sample, which is analogous to the plankton that is highly concentrated across the *d* axis. The following equation determines the weight element, or quantity *α*.18
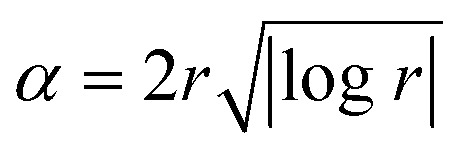


Manta rays construct a lengthy chain-like framework and swim in a spiral motion to find food during cyclone foraging. An example of the agent's update procedure is as follows:19
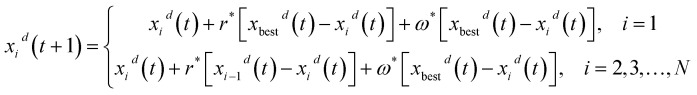
20
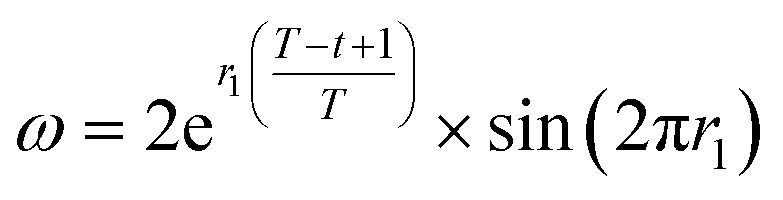


The following is a representation of this procedure. The agents are required to move a random step away from their present location in order to investigate an alternate location. They can investigate the search space autonomously of the optimal agent since this arbitrary distance is established by utilizing a variable, as shown:21
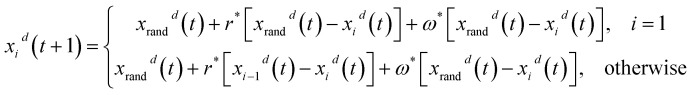
In the existing illustration, *X* stands for a particular entity in the iteration space, and its characteristics are as follows:

22 *x*_rand_^*d*^(*t*) = LB^*d*^ + rand(UB^*d*^ − LB^*d*^)

The placements of samples are subsequently mathematically described in the somersault foraging procedure improvements as follows:

23 *x*_*i*_^*d*^(*t* + 1) = *x*_*i*_^*d*^(*t*) + ∅*[*r*_2_*x*_best_^*d*^(*t*) − *r*_3_*x*_*i*_^*d*^(*t*)], *i* = 1, 2, 3, …, *N*

In this case, *r*_2_ and *r*_3_ can have any value inside the given range [0,1].

MRFO was chosen for the intelligent optimization of the current study due to potentially advantageous performance benefits, which include rapid convergence and better exploration–exploitation equilibrium compared to other metaheuristic algorithms inspired by biological neurons. MRFO was implemented in Python using MEALPY library.

## 3. Results and discussion

### 3.1. Optimal formulation and characterization of ternary oil feedstock

Table 3 shows the data from most important variables obtained from the analysis of variance (ANOVA). ANOVA estimated *p*-value and *F*-value ratios are crucial metrics for assessing the applicability and precision of quadratic models given in eqn (24)–(26). Each parameter featuring a *p*-value < 5% (*p*-value < 0.05) were considered relevant parameters in the model; given that the likelihood of *p*-value was examined at an assurance level > 95%.^2,33^ Consequently, according to Table 3, all input variables were relevant in predicting the investigated physicochemical properties of MNEO. In addition to *p*-values, the relevance of each term in the quadratic model was evaluated using the corresponding magnitudes of *F*-values. This was accomplished by analyzing how each model's residual and pure error interacted with the related lack of fit. From the information in Table 3, and *F*-value of 378.3, 47.06, and 910.8 highlights the relevance of the quadratic models in predicting the acid value, density and iodine value, respectively of MNEO, relative to pure error. Furthermore, lack of fit *F*-values of 0.4491 (acid value), 0.1434 (density), and 8.12 (Iodine value) demonstrated the insignificant impact of the lack of fit in describing the physicochemical properties of MNEO. *R*^2^ (AV = 0.9921; *ρ* = 0.9058; IV = 0.9978) and adjusted-*R*^2^ (AV = 0.9920; density = 0.8587; IV = 0.9967) were satisfactorily correlated, given that the numerical difference between each of them is not greater than 0.2. It is important to note that the agreement between *R*^2^ and adjusted-*R*^2^ indicates a satisfactory correlation between experimental and predicted values as demonstrated in Table S1. Other statistical appraisal indices including adequacy precision (APR), coefficient of variance (CV) authenticated the reproducibility and applicability of the mixture design modeling of MNEO. The results obtained here give credence to the fact that the formulated oil blend actually possess the properties obtained in this study.

24 Acid value = 0.07371*x*_1_ + 0.352519*x*_2_ + 0.036774*x*_3_ − 0.001930*x*_1_*x*_2_ + 0.000659*x*_1_*x*_3_ − 0.00114*x*_2_*x*_3_

25 Density = 0.008795*x*_1_ + 0.008606*x*_2_ + 0.009053*x*_3_ − 0.0000005212*x*_1_*x*_2_ + 0.0000017947*x*_1_*x*_3_ − 0.0000008884*x*_2_*x*_3_

26 Iodine value = 0.5186*x*_1_ + 0.25232*x*_2_ + 0.809505*x*_3_ − 0.005021*x*_1_*x*_2_ + 0.002527*x*_1_*x*_3_ − 0.008564*x*_2_*x*_3_

where *x*_1_, *x*_2_, and *x*_3_ represent the proportions of WCO, RO-POME, and CO in the blend.

**Table 3:** Test of significance for model coefficients and analysis of variance

Acid value						Density					Iodine value				
Source	SS	d*f*	MS	*F*-Value	*p*-Value	SS	d*f*	MS	*F*-Value	*p*-Value	SS	d*f*	MS	*F*-Value	*p*-Value
*X*_1_ *X*_2_	25.13	1	25.13	54.09	<0.0001	1.83 × 10^−6^	1	1.83 × 10^−6^	0.12	0.0337	170.14	1	170.14	243.97	<0.0001
*X*_1_ *X*_3_	3.70	1	3.7	7.97	0.0181	0.000011	1	0.000011	1.83	0.0205	54.46	1	54.46	78.08	<0.0001
*X*_2_ *X*_3_	9.25	1	9.25	19.91	0.0012	5.88 × 10^−6^	1	5.88 × 10^−6^	0.39	0.0485	546.82	1	546.82	784.07	<0.0001
Mixture	841.92	2	420.96	905.85	<0.0001	0.0014	5	0.0003	19.24	<0.0001	2425.31	2	1212.65	1738.79	<0.0001
Model	879	5	175.8	378.30	<0.0001	0.0014	2	0.0007	47.06	<0.0001	3176.02	5	635.2	910.80	<0.0001
Lack of fit	2.38	7	0.34	0.45	0.8274	0	7	5.36 × 10^−6^	0.14	0.9835	6.62	7	0.95	8.12	0.0565
Residual	4.65	10	0.46			0.0001	10	0			6.97	10	0.70		
Pure error	2.27	3	0.76			0.0001	3	0			0.35	3	0.12		
*R* ^2^ = 0.9947						*R* ^2^ = 0.9058					*R* ^2^ = 0.9978				
Adjusted *R*^2^ = 0.9921						Adjusted *R*^2^ = 0.8587					Adjusted *R*^2^ = 0.9967				
Adequacy precision = 75.6360						Adequacy precision = 18.8360					Adequacy precision = 108.9533				
CV = 5.2						CV = 0.4394					CV = 1.29				
Mean = 13.10						Mean = 0.8807					Mean = 64.65				

**Fig. 2:**
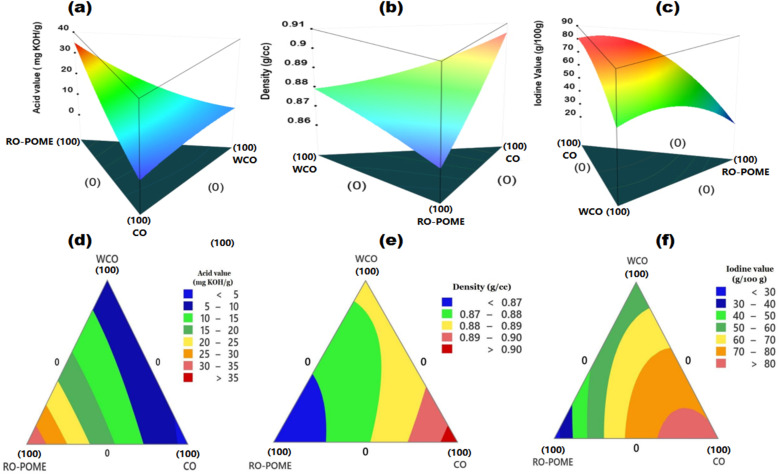
Surface and contour plots for effect of mixing ratio on acid value (a and d), density (b and e), and iodine value (c and f) for optimal formulation of MNEO feedstock.

Based on the formulated models (eqn (24)–(26)), optimized blends of nonedible oils were formed, and the associated surface and contour plots are given in Fig. 2. An overview of the surface and contour plots shows the synergistic importance of blending the feedstock. This is highlighted by the fact that each of the studied response variable is associated with an elevated concentration (>70%) of either species. Specifically, an acid value of 23.72 mg KOH per g was obtained at blending ratios of WCO (11.99%), RO-POME (71.1%), and CO (16.91%), while an iodine value of 80.47g I_2_/100 g oil was obtained at 18.06%, 9.55%, and 72.40% of WCO, RO-POME, and CO, respectively. This trend was consistent with the observation noted for density and viscosity parameters, where significant concentrations of CO resulted in elevated their values in MNEO product. According to Godswill *et al.*,^67^ the significant acidity in palm oil based RO-POME is attributed to the hydrolysis of triacylglycerols into free fatty acids. The authors highlighted that this hydrolysis process is often triggered during the palm oil processing step, and accelerated by lipase enzyme contained in the POME slurry. Similarly, ricinoleic acid, and its associated hydroxyl group which constitutes more than 90% of castor oil has been noted to be responsible for elevated densities in the sample.^68^

Table 4 shows the optimization data for achieving optimal formulation of MNEO, while Table 5 presents relevant physicochemical properties of each oil species and optimized samples of MNEO. According to Table 4, optimization criteria for all factors were kept “in range”, implying that boundary conditions for optimization procedure included all factor levels within the upper and lower bounds. Similarly, the optimization criteria for selected responses were set minimization goal, considering the fact that elevated values of any of the studied responses is undesirable for biodiesel feedstock.^17^ At the end of numerical optimization, the acid value, density, and iodine value were 9.018 mg KOH per g, 0.931 kg m^−3^, and 68.533 g I_2_/100 g oil, respectively, at a composition of 21.31% WCO, 18.45% RO-POME, and 60.24% CO.

**Table 4:** Optimization levels for formulation of MNEO species

Scope	Variables	Optimization criteria	Value
Input parameters	WCO (%)	In range	21.31
	RO-POME (%)	In range	18.45
	CO (%)	In range	60.24
Responses	Acid value (mg KOH per g)	Minimize	9.018
	Density (g cm^−3^)	Minimize	0.9031
	Iodine value (g I_2_/100 g oil)	Minimize	68.533

**Table 5:** Selected physicochemical properties of single and optimally formulated MNEO species

Property	Castor oil	RO-POME	WCO	MNEO
Acid value (mg KOH per g oil)	4.110	34.830	8.010	9.018
FFA (%)	1.812	17.730	4.042	4.404
Viscosity @ 28 °C (m. Pas)	154.680	27.660	54.326	109.857
Saponification value	180.753	194.000	186.656	184.455
Iodine value (g I_2_/100 g oil)	80.920	24.840	51.830	68.533
Average molecular wt (g mol^−1^)	928.500	839.870	896.000	898.140
Density @ 28 °C (g cm^−3^)	0.965	0.902	0.938	0.903
Specific gravity	0.983	0.918	0.955	0.920

The physicochemical properties of single and blended nonedible oil species were characterized and presented in Table 5. According to the result in Table 5, it is evident that blending the oil species contributed significantly in improving the physicochemical of MNEO. For instance, CO, having an acceptable FFA of 1.812 contributed significantly in reducing the FFA of RO-POME (17.730%) to produce a MNEO having an FFA of 4.404% which is comparable to the values reported in other works.^69,70^ Also, an undesirable high viscosity of 154.68 m Pas in CO was reduced to 109.857 m Pas in MNEO, following addition of RO-POME and WCO. Consequently, judging from the improved properties of the blended oil feedstock, it will be a more desirable biodiesel feedstock compared to each of the oils in transesterification reaction.

Using gas chromatography in conjunction with mass spectroscopy, the fatty acid profile of MNEO was examined. The peaks were identified by comparing their mass spectrum and retention time using a mass spectra database, and the results are presented in Table 6. According to the values in Table 6, the quantitative order of fatty acids present in MNEO are oleic acid (30.227%), linoleic acid (19.571%), stearic acid (16.486%), and palmitic acid (11.049%). These composition reflects a desirable mixture of saturated and unsaturated fatty acids in MNEO suitable for biodiesel reaction.^71,72^ According to Lanjekar *et al.*,^71^ the synthesis of biodiesel requires an appropriate ratio of saturated and unsaturated fatty acids. The authors stated that although saturated fatty acids can promote the prevention of NO_*x*_ emissions, and improve oxidative stability, they can also cause unsatisfactory cold flow characteristics. Also, it is important to highlight that although polyunsaturated fatty acids enhance cold flow characteristics, they have been found to be responsible for increasing NO_*x*_ emissions and declined oxidative stability.^72^ Therefore, MNEO containing 29.96%, and 64.69% of saturated and unsaturated fatty acids, respectively, illustrate a desirable feedstock with good potentials of producing a biodiesel having good combustion characteristics, oxidative stability, and cold flow properties.

**Table 6:** Fatty acid profile of mixed non-edible oils

Fatty acid	RT	Molecular wt (g mol^−1^)	Amount (%)	Nature
Palmitic acid (C16 : 0)	5.884	256.43	11.0486	Saturated
Stearic acid (C18 : 0)	6.124	284.48	16.4856	Saturated
Behenic acid (22 : 0)	15.026	340.58	2.4241	Saturated
Total saturated acids	—	—	29.9583	Saturated
Oleic acid (C18 : 1)	7.240	282.46	30.2267	Monounsaturated
Total monounsaturated acids	—	—	30.2267	Monounsaturated
Linoleic acid (C18 : 2)	9.618	280.4472	19.5714	Polyunsaturated
Linolenic acid (C18 : 3)	10.793	278.43	4.1378	Polyunsaturated
Arachidonic acid (C20 : 4)	13.522	304.47	2.7470	Polyunsaturated
Eicosapentaenoic acid (C20 : 5)	15.362	302.451	3.0530	Polyunsaturated
Docosahexaenoic acid (C22 : 6)	17.600	328.488	4.9557	Polyunsaturated
Total polyunsaturated acids	—	—	34.4649	Polyunsaturated
Others	—	—	5.3501	—
Total saturated fraction	—	—	29.9583	Saturated
Total unsaturated fraction	—	—	64.6916	Unsaturated

### 3.2. Catalyst characterization

#### 3.2.1. FTIR

**Fig. 3:**
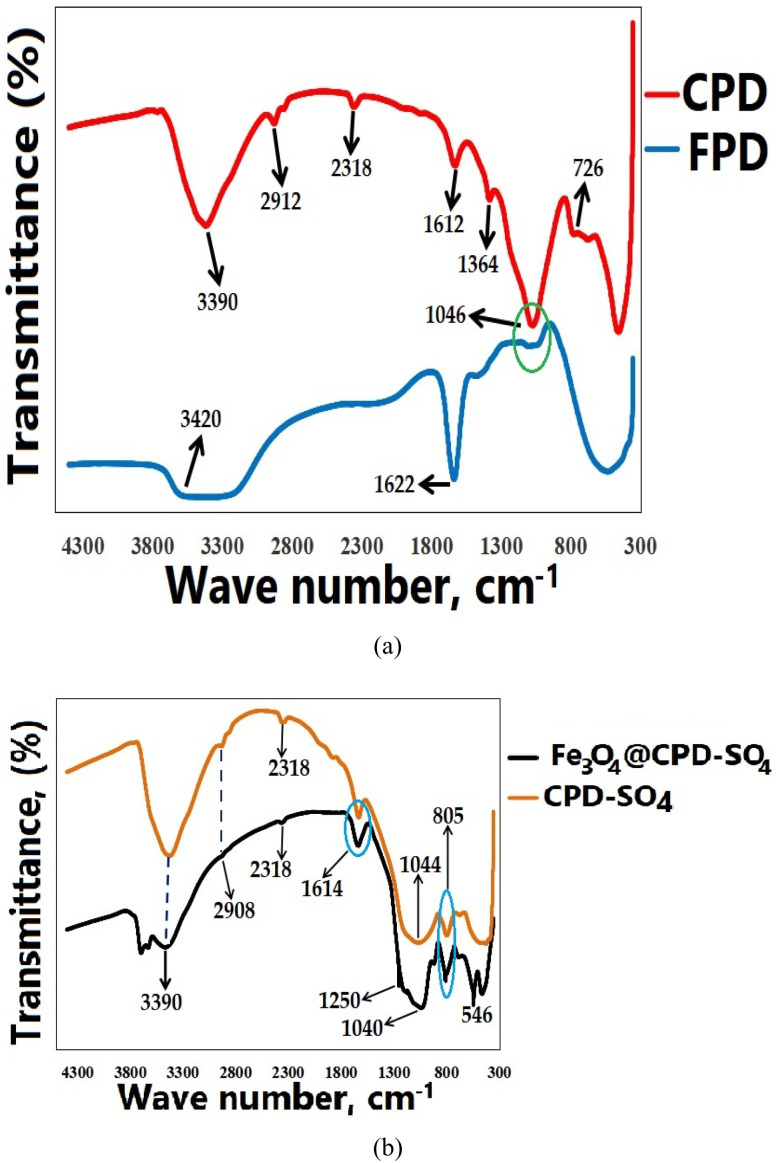
FTIR spectrum of (a) FPD and CPD, (b) CPD–SO_4_ and Fe_3_O_4_@CPD–SO_4_ catalyst.

FTIR spectra of all catalyst species preparations steps is depicted in Fig. 3. According to the result in Fig. 3(a), three prominent peaks were detected including 3420 cm^−1^, 1622 cm^−1^, and 1046 cm^−1^. The broadband in fresh poultry droppings (FPD) detected in 3420 cm^−1^ drastically reduced to a sharp peak in waveband 3390 cm^−1^ following calcination (CPD in Fig. 3(a)). The slight shift in wave number and significant transformation from broadband to a sharp peak in calcined poultry droppings (CPD) underscores the unstable nature of hydroxyl groups at elevated temperatures.^73^ The presence of carbonyl and carboxyl groups in carbohydrates found in dietary cellulose (FPD in Fig. 3(a)), and the presence of C–O stretching were expose by vibrational wave number at 1622 cm^−1^ and 1046 cm^−1^, respectively. These functional groups are typically associated with organic wastes containing significant amounts of fibrous materials.^74^ Given their large surface area and porous nature, which offer a large number of active sites and facilitate mass transport and substrate accessibility for effective transesterification, fibrous substances are crucial for biodiesel catalysis.^75^

Similarly, C–O stretching of polysaccharides which initially appeared as a weak, slightly broad peak, transformed to a strong sharp stretching vibration in 1612 cm^−1^ after calcination. Four new peaks were detected after calcination, out of which three peaks (2912 cm^−1^, 1364 cm^−1^, 726 cm^−1^) were useful in describing the calcined poultry droppings (CPD in Fig. 3(a)). Aliphatic CH_*n*_ vibration was detected in wave number 2912 cm^−1^. This aliphatic CH_*n*_ vibration showcases the breaking of weak single bonds between carbon and hydrogen of alkyl groups. This bond breaking phenomenon is a consequential reaction associated with thermal activation. The finger print region highlights two peaks, including 1364 cm^−1^ and 726 cm^−1^ signaling the presence of CH_2_ unit biopolymers and wagging vibrations of CH bond in aromatic and hetero-aromatic compounds. In catalyst development, heat activation is crucial for separating weak bonds as it enables molecular reconfiguration to create an ideal structure in the final catalyst, builds active sites, and facilitates a mechanism for transesterification reaction to take place at greater efficiency and less energy.^76^

Vibrational spectroscopy of sulfonated CPD and magnetic catalyst (Fe_3_O_4_@CPD–SO_4_) in Fig. 3(b) did not show significant deviations in surface structure from the result obtained in CPD. The vibrational position of most wave numbers was retained, while a few produced minor shifts after activation. For instance, wave numbers 3390 cm^−1^, and 2908 cm^−1^ did not shift from the original positions after sulfonation and ferromagnetic activation. Also, the wavenumber at 2318 cm^−1^ which indicates the presence of C≡N vibration associated with nitriles and isocyanates, was not deformed after sulfonation and magnetization. The sulfonation and ferromagnetic activation processes were validated by the appearance of wavenumbers 805 cm^−1^ (sulfonated polysaccharides), 1250 cm^−1^ (sulfonated polymeric aromatic compounds), and 546 cm^−1^ (Fe–O).^1,77^ The FTIR spectrum of Fe_3_O_4_@CPD–SO_4_ highlights a catalyst with the potentials to propagate esterification and transesterification reactions through an energy efficient means. In additions to these properties, the catalyst is susceptible to magnetic recovery. These advancements in catalytic properties are as a result of changes associated with precursor (FPD) transformation during Fe_3_O_4_@CPD–SO_4_ activation.

#### 3.2.2. SEM-EDX

**Fig. 4:**
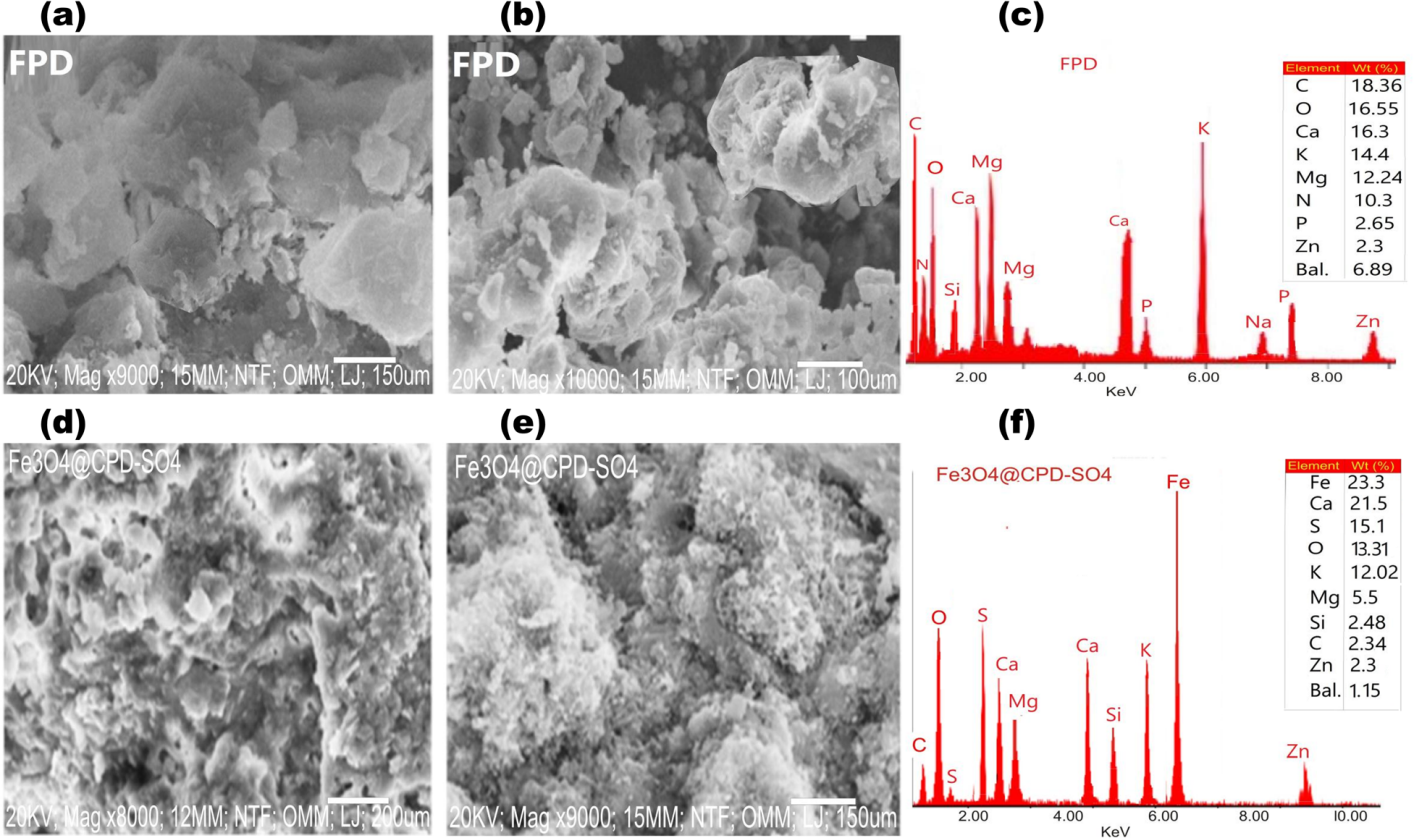
SEM-EDX of (a–c) fresh poultry droppings and (d–f) Fe_3_O_4_@CPD–SO_4_ catalyst.

The morphological features along with the Energy dispersive X-ray analysis (EDX) of FPD and derived catalyst (Fe_3_O_4_@CPD–SO_4_) at different magnifications is depicted in Fig. 4. According to Fig. 4(a) and (b), the morphological image of FPD showcases an assemblage of roughly shaped flakes, overlapping several layers of irregular platelets. These observations are consistent with FTIR results which implied that FPD contained fibrillary structure and crispy rough materials.^78^ Overlapping layer frameworks are crucial for transesterification catalysts given that they can improve stability, which promotes longevity and reusability.^79^

SEM micrograph Fe_3_O_4_@CPD–SO_4_ (Fig. 4(d and e)) depicts a surface having partly spatial distribution of uniform pseudo-spherical particles. Further observation reveals the appearance of a porous dark matrix in Fe_3_O_4_@CPD–SO_4_, implying a significant enlargement in particular pore diameter compared to FPD. These observations noted in Fe_3_O_4_@CPD–SO_4_ are consistent with the surface of a catalyst possessing improved morphological properties with the potentials of accelerated catalyzes of transesterification reaction compared to FPD.^80^

The EDX spectroscopy showing elemental distribution on the surface of FPD and Fe_3_O_4_@CPD–SO_4_ were listed in Fig. 4(c) and (f), respectively. From the average elemental distribution of FPD, the major elements contained in FPD include carbon (18.36%), oxygen (16.55%), calcium (16.03%), potassium (14.40%), magnesium (12.24%) and nitrogen (10.30%). These elemental compositions are associated with animal wastes containing fibro-proteinous substrates and uric acids, which make up a significant portion of poultry droppings. After passing through the physicochemical tripod activation steps of calcination, sulfonation and magnetization, the elemental make up of Fe_3_O_4_@CPD–SO_4_ was significantly modified. Major elemental make up of Fe_3_O_4_@CPD–SO_4_ includes iron (23.3%), calcium (21.5%), sulfur (15.1%), potassium (12.02%). The increase in amounts of calcium and corresponding reduction in carbon and oxygen is attributed to thermal activation step, while the presence of Fe and S validated the magnetization and sulfonation steps, respectively. The distribution of elements on the surface of Fe_3_O_4_@CPD–SO_4_ underscores the potentials of this catalyst species in propagating one-pot transesterification reaction *via* bi-functional mechanism.

#### 3.2.3. XRD

**Fig. 5:**
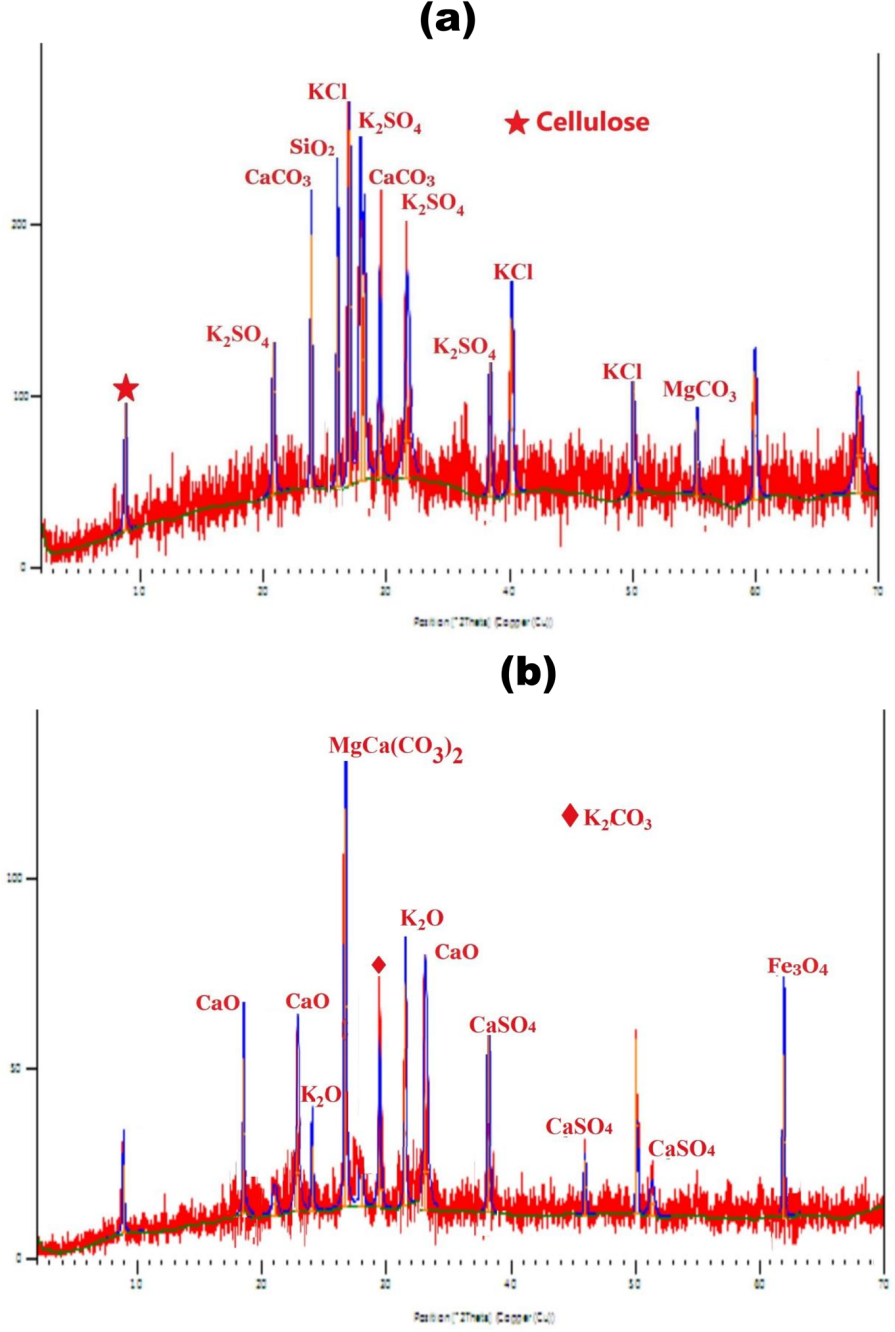
XRD crystallography of (a) FPD (b) Fe_3_O_4_@CPD–SO_4_ catalyst.

**Fig. 6:**
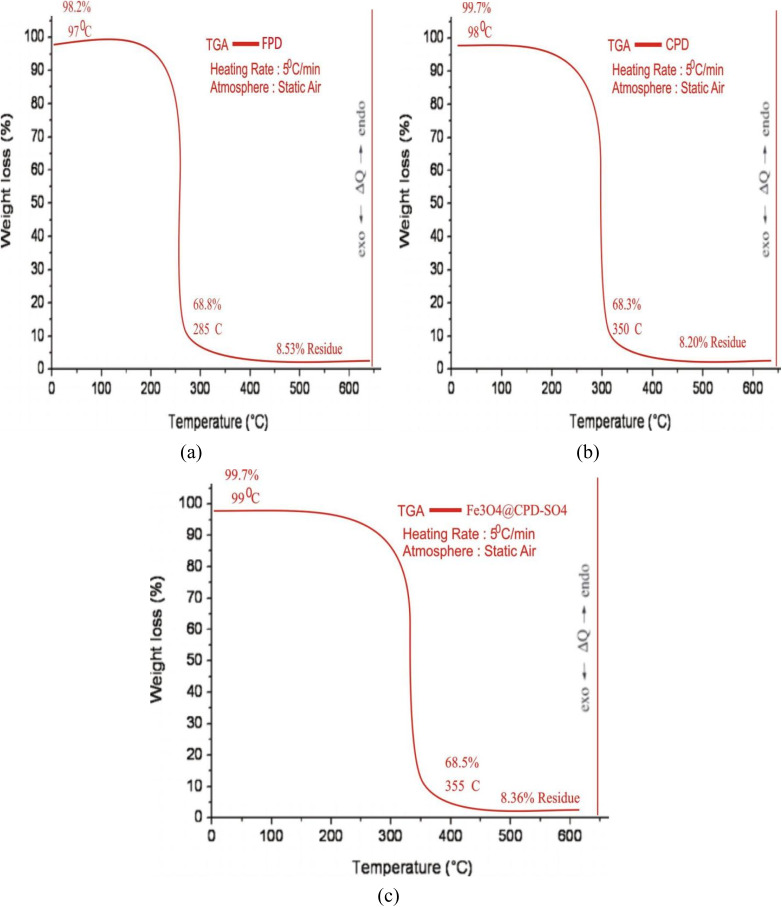
TGA result of (a) FPD, (b) CPD, and (c) Fe_3_O_4_@CPD–SO_4_ catalyst.

Xray diffraction was used to explore the crystallinity of FPD and Fe_3_O_4_@CPD–SO_4_. Crystalline polymorph of FPD (Fig. 5(a)) demonstrated a well resolved absorption spectrum with sharp reflections illustrating the presence of dietary cellulose, silica (SiO_2_), carbonate forms of calcium and magnesium, including chlorinated and sulfonated potassium. The presence of these compounds could be attributed to the dietary make-up of the poultry birds which are also contained in their excreta.^81,82^ Furthermore, the detection of cellulosic crystals correlates favorably with the observations noted in FTIR and SEM that FPD contain dietary cellulose. The XRD of Fe_3_O_4_@CPD–SO_4_ show the formation of new crystalline structures in Fig. 6(a). The formation of CaO at 2*θ* = 18.6°, 23.1° is attributed to the thermal transformation of CaCO_3_ to stable oxide of calcium (CaO). Furthermore, it is also discernible to note that high intensity peaks at 2*θ* = 24.1° and 27.8° highlighting the crystalline presence of CaCO_3_ in FPD, completely disappeared in the crystalline polymorph of Fe_3_O_4_@CPD–SO_4_, underscoring the impact of calcination in crystalline transformation of FPD. Furthermore, a spread of calcium sulfonate (CaSO_4_) crystals in the synthesized catalyst (Fig. 5(b)) may be attributed to the interactions between thermally stable CaO from CPD and sulfuric acid during chemical activation. It is also important to note that the ferric property of Fe_3_O_4_@CPD–SO_4_ was exposed by the presence of Fe_3_O_4_ at 2*θ* = 62.1°. These results correlate favorably with the observations of other researchers who synthesized magnetic catalyst for transesterification reaction.^83^ Given that CaO (a well-known Lewis base),^84^ and acid containing sulfonate groups of CaSO_4_ are present in Fe_3_O_4_@CPD–SO_4_ catalyst, it implies that the final catalyst possess the potentials of inducing both acidity and alkalinity required for esterification and transesterification reactions, respectively.

#### 3.2.4. TGA

**Fig. 7:**
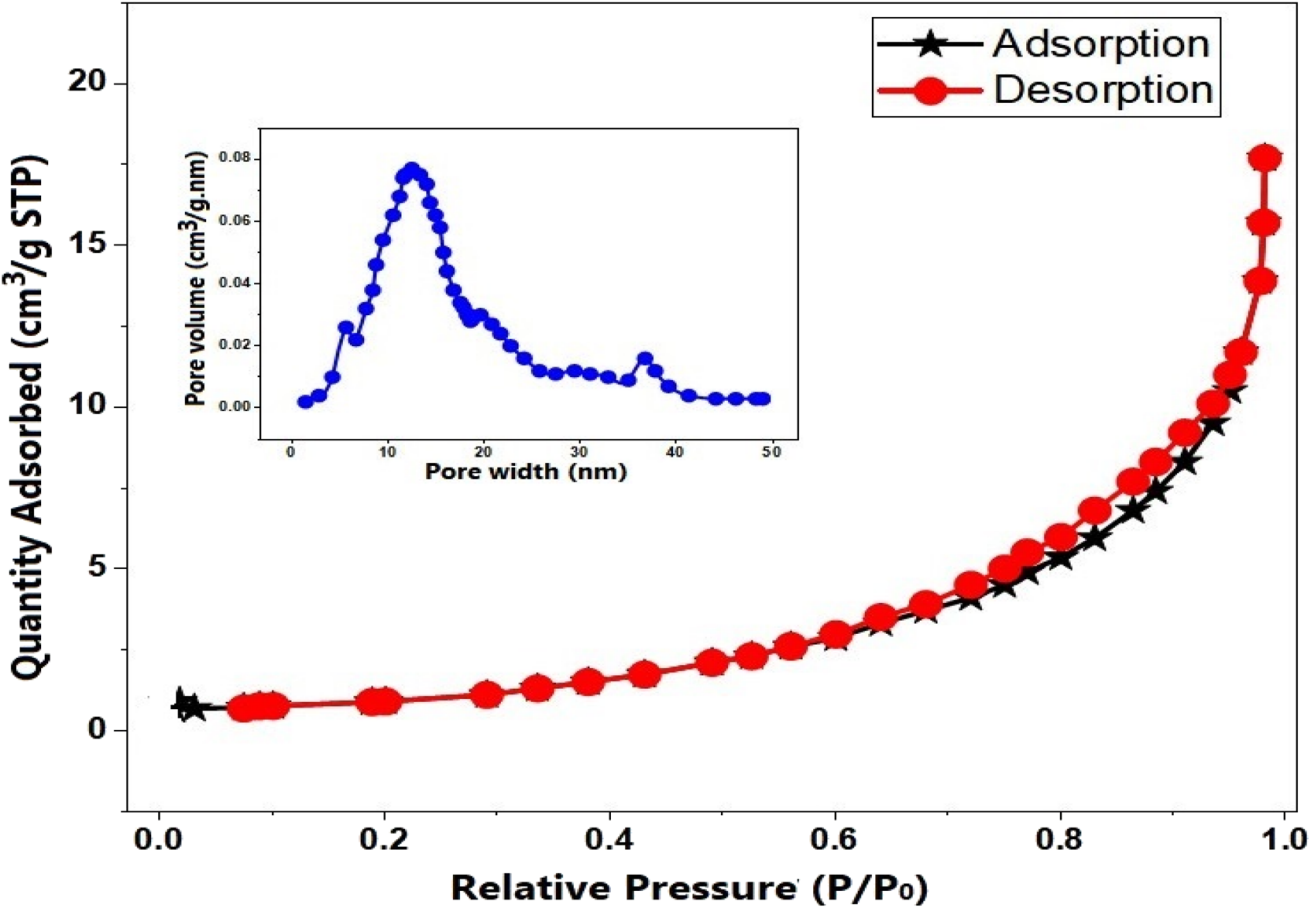
N_2_ adsorption–desorption isotherms of Fe_3_O_4_@CPD–SO_4_.

Thermo-gravimetric analysis (TGA) was used to explore the physicochemical changes in the samples and establish the thermal stability of FPD, CPD and Fe_3_O_4_@CPD–SO_4_ at each preparation stage. These results are presented in Fig. 6. According to Fig. 6(a), thermal decomposition of FPD and catalyst species occurred in three phases. The first phase of decomposition occurred from 0 °C to 97 °C to 99 °C. During this stage, a significant dehydration occurs leading to loss of moisture and surface volatile nutrients.^85^ At the end of this stage, FPD, CPD (Fig. 6(b)), and Fe_3_O_4_@CPD–SO_4_ (Fig. 6(c)), retained 98.2%, 99.7%, and 99.7% of their initial weights, respectively. The second stage of decomposition demonstrated an accelerated rate of weight loss occurring between 97 °C and 285 °C for FPD (Fig. 6(a)), and between 98 °C and 350 °C, and 99 °C and 355 °C for CPD (Fig. 7(b)) and Fe_3_O_4_@CPD–SO_4_ (Fig. 6(c)), respectively. This second stage accounted for 68.8% wt. loss in FPD (Fig. 6(a)), and 68.3 wt% loss of CPD (Fig. 6(b)), while Fe_3_O_4_@CPD–SO_4_ (Fig. 6(c)) recorded a 68.5% weight loss. It is also interesting to note that despite the fact that upper limit of decomposition temperature occurring in Fe_3_O_4_@CPD–SO_4_ (Fig. 6(c)) was 70 °C above FPD (Fig. 6(a)), the corresponding weight loss during this period was approximately identical, this underscores the superior thermal stability of Fe_3_O_4_@CPD–SO_4_ (Fig. 6(c)) catalyst compared to FPD (Fig. 6(a)) precursor. The third stage of decomposition was between the upper limit of the second stage and termination temperature of 600 °C.

Given that a catalyst exhibiting significant thermal stability is capable of withstanding the elevated temperatures needed for the process to take place, it is preferred for transesterification. This prevents degeneration and guarantees steady catalytic performance throughout the process.

#### 3.2.5. Surface area and textural analyses

N_2_ adsorption–desorption technique was employed to estimate the textural features of Fe_3_O_4_@CPD–SO_4_ catalyst species. The results of this analyses are presented in Table 7 and Fig. 7. From the graphical illustration, Fe_3_O_4_@CPD–SO_4_ catalyst depicts a blend of Type-1 and Type-IV IUPAC classified adsorption isotherm with H4 hysteresis loop, authenticating the presence of microporous and mesoporous materials.^86,87^ According to the numerical values in Table 7, BET surface area of Fe_3_O_4_@CPD–SO_4_ is 10.864 m^2^ g^−1^ while the pore volume and pore diameter were 0.03136 m^3^ g^−1^ and 15.346 nm, respectively. According to Sahar *et al.*,^86^ both microporous and mesoporous catalysts provide several benefits, such as a wide surface area, tunable pore diameters, and good heat durability, all of which improve reactant permeability and reaction effectiveness. These features correlate favorably with the observations noted during the FTIR analysis where the textural properties were linked.

**Table 7:** Textural characteristics and surface area analysis

BET specific surface area (m^2^ g^−1^)	Pore volume (m^3^ g^−1^)	Pore diameter (nm)
10.864	0.03136	15.346

### 3.3. ML modelling for biodiesel synthesis

The results of experimental and machine learning modeling of the present system were supplied in Table S2 of SI, while the optimized hyperparameter data of each model was given in Table S3 (SI). The optimized hyperparameter data were useful in evaluating the training and test performance of the ML algorithms.

#### 3.3.1. Hyperparameter tuning

Support vector regression (SVR) model was developed using radial basis K-fold cross validator parameter. A five-fold cross-validation parameter was selected owing to the fact that it produced the least iteration time and estimation error, which are important signals validating the non-existence of model over-fitting. The total time employed by the vector regression to perform training_time split algorithm over a 42 random state was 238 seconds. At the end of training_split algorithm, the model complexity/error trade-off signal (*C*), error tolerance margin (*ε*), and influence of training parameter (*γ*), were 1.0635, 0.4154, and 0.0963, respectively. These parameters authenticate the reliability of SVR model performance. For instance, high levels of *C* produce a model which tries to replicate the training information as closely as possible, and it more susceptible to individual data values, potentially ending up in over-fitting, while low values of *C* leads to model under-fitting. According to Su *et al.*,^88^ the desirable range for *C* is typically 1 ≤ *C* ≤ 10. Consequently, a *C* value of 1.0365 highlights an efficiently reliable model capable of replicating experimental values withing the design space while not suffering over-fitting. Table S3 contains a detailed data of training and test hyperparameter for ANN algorithm. Regularization parameter, learning rate, scoring metric, and cross-validation were useful hyperparameters employed to assess the stability of ANN. Similarly, extreme gradient boosting (XGB) machine learning algorithm was designed using *k*-fold cross validator. This algorithm exploits gradient boosting approximation functions to achieve high precision prediction rates and associated minimized over-fitting drawbacks. The main identified optimum hyper-parameters include max_depth (10), sub-sample (0.82), min_child_weight (3.0), n_estimates (1400) and regularization strengths (Reg_*α*: 0.06746; Reg_*λ*: 0.04349). These parameters, most importantly Reg_*α* and Reg_*λ* regularization strengths satisfactorily emphasized the fact that XGB algorithm modeling technique was not associated with the risk of over-fitting during the iteration process.^65^ Other hyper-parameters were selected as follows: learning rate = 0.012; *γ* = 0.0004; colsample_bytree = 0.9; random_state = 84.

#### 3.3.2. Model performance assessment

The performance of each modeling technique was assessed using analytical indices such as *R*^2^, MSE, RMSE, and AIC, the actual numerical significance of their performance was expressed. According to information available in literature, the performance of a predictive model is reasonably adequate if *R*^2^ is greater than 0.9.^89^ According to the information in Table 8, the range of the full data *R*^2^ values was 0.9480 ≤ *R*^2^ ≤ 0.9644, emphasizing the robust ability of each model to efficiently predict the nonlinear dynamics of MNEO transesterification process. Explicitly typifying this is to understand that SVR model, having an *R*^2^ value of 0.9480 implies that 94.8% of the experimental data can accurately be predicted by SVR algorithm. This same principle applies to ANN (95.95) and XGB (96.44%). MSE provides a basic indicator of how effectively a model explains the data by calculating the average squared variance across estimated and actual measurements. The values of MSE were found to be significantly low (<0.1) underscoring the applicability of each technique in modeling the nonlinear nature of transesterification reaction of the present system. The mean size of discrepancies through expected and actual values is measured by RMSE. An overview of the MSE and RMSE values indicate that XGB marginally performed better than ANN and SVR. The Akaike's information criterion (AIC) was further used for model discrimination. Small magnitudes of AIC, like the values obtained for XGB (22.0692) were preferred over the values of ANN (76.2154) and SVR (78.7082) because they showed that the XGB had an acceptable balance involving complexity and precision. A generic assessment of the hybrid error appraisal established that XGB performance, although not significantly, was superior to ANN and SVR in capturing the nonlinear nature of the present system. Consequently, the accuracy of model predictions followed the order XGB > ANN > SVR. Given that XGB performed better than other models, it was adopted for SHAP and MRFO analyses.

**Table 8:** Fitness metrics for ML models

Parameter	Value		
	ANN	SVR	XGB
Train *R*^2^	0.9880	0.9562	0.9705
Test *R*^2^	0.8221	0.9061	0.9353
Full data *R*^2^	0.9595	0.9480	0.9644
MSE	0.0405	0.0520	0.0356
RMSE	0.2012	0.2280	0.1887
AIC	76.2154	78.7082	22.0692

#### 3.3.3. Intelligent optimization

Table 9 shows the intelligent optimization data obtained from XGB-coupled MRFO algorithm (XBG-MRFO), along with the associated hyperparameter values. To regulate the technique's behavior, hyperparameters typically pre-set. These hyperparameters affect the algorithm's search-exploitation stability, converging rate, and capacity for minimizing local optima. Essential hyperparameters such as population size and somersault range were appropriately set as 10 and 1.0, respectively, which were sufficient enough to stabilize the capacity of the foraging algorithm to, locate global optima.^90^ Applying the MRFO algorithm, optimum variable levels of temperature 50 °C, catalyst dosage 3.01 wt%, methanol-oil ratio 30, and reaction time 2.40 h were obtained as shown in Table 9. At these optimum values, a predicted biodiesel yield of 99.13% was obtained. A triplicate validation experiment was performed at the predicted optimum variable parameters, after which biodiesel yield of 98.16% was obtained, demonstrating a high reliability in the modeling and optimization techniques applied in this study.

**Table 9:** Intelligent optimization data

Feature		Hyper parameter		Optimized output	
Parameter	Value	Parameter	Value	Predicted	Validated
Molar ratio	30.0	Epoch	5	99.68%	98.16%
Temperature	50 °C	Pop_size	10		
Time	2.4 h	Somersault_range	1.0		
Catalyst dosage	3.01 wt%				

Table S4 presents a comparative performance of our findings to the most recent publications in transesterification reaction of blended oils over solid catalysts. An overview of these results reveals insightful information regarding the performance of Fe_3_O_4_@CPD–SO_4_. The optimum yield of our catalyst (98.16%) outperformed dolomite based catalyst (87.7%) reported by Vieira *et al.*,^91^ in transesterification of mixed castor and cotton seed oil. Worthy of note is the fact that the obtained values here aligned closely with the findings of Babatunde *et al.*,^92^ (97.67%), Boro *et al.*,^93^ (96.57) and Basumatary *et al.*,^94^ (96.34%). Despite these strong correlations, the significant catalytic performance of Fe_3_O_4_@CPD–SO_4_ was exemplified by the fact that a lower amount (3.01 wt%) was employed to achieve a comparable yield as opposed to 15 wt% (Areca nut leaf ash–K_2_CO_3_) and 9 wt% (Banana waste), reported by Boro *et al.*, (2024) and Basumatary *et al.*, (2024), respectively. The dependability of the proposed strategy is demonstrated by the fact that the described ideal conditions found in this work matched those found in previous studies and, in many cases, even exceeded them.

#### 3.3.4. Input–output visualization

**Fig. 8:**
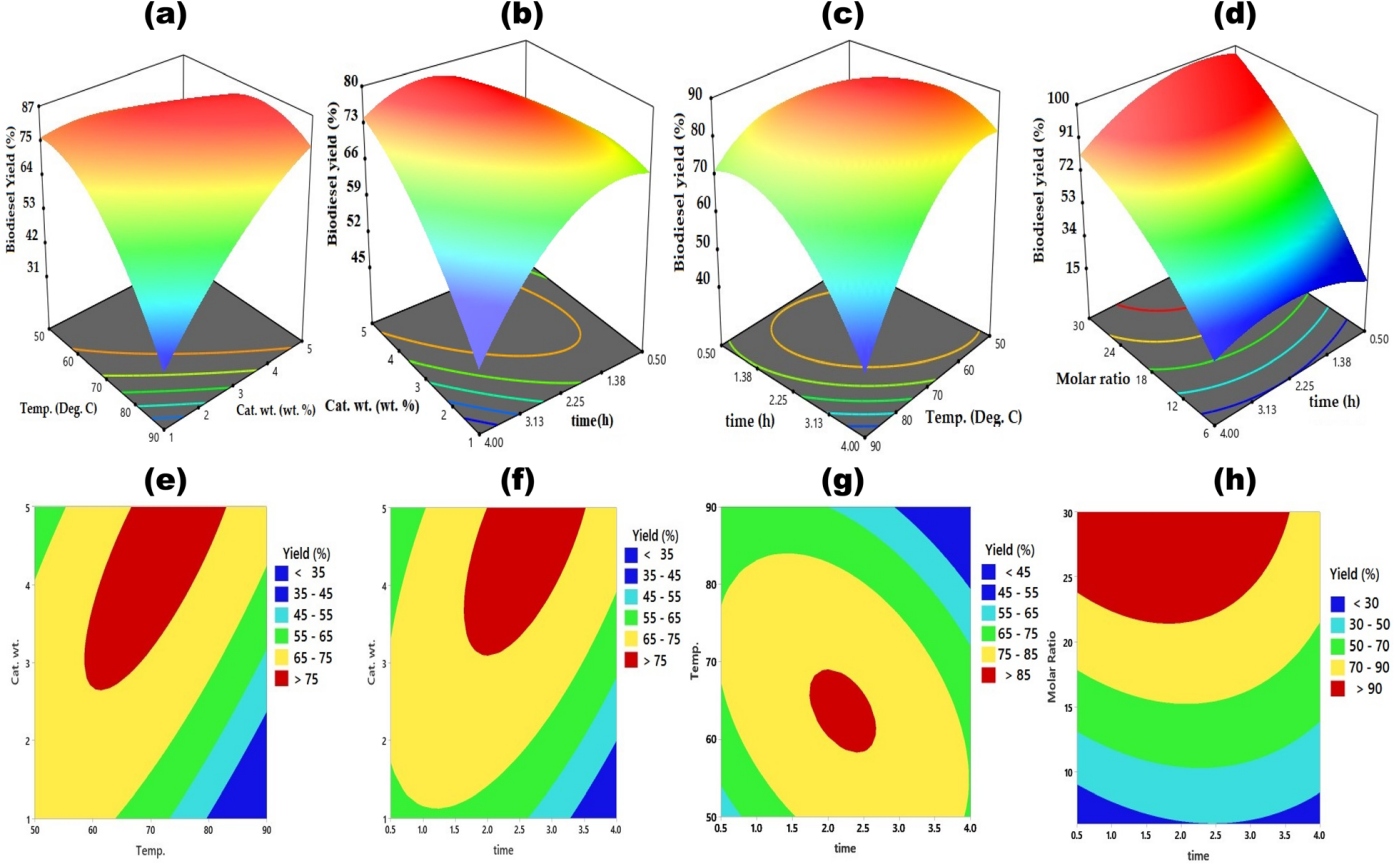
Influence of process parameters on transesterification of MNEO using Fe_3_O_4_@CPD–SO_4_ catalyst.

**Fig. 9:**
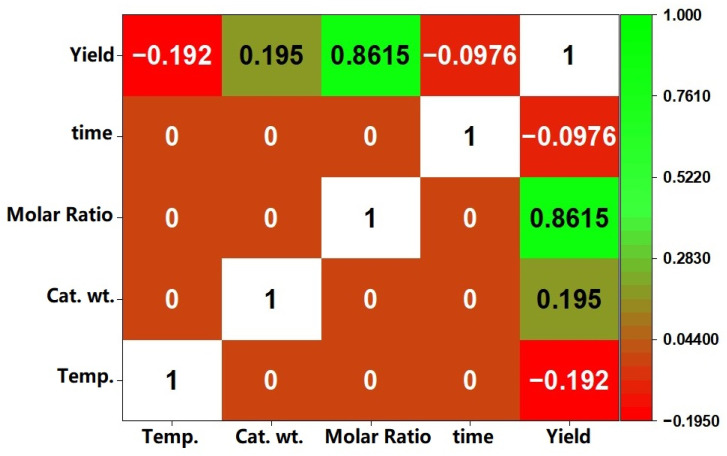
Pearson heat-map correlation data.

Fig. 8 depicts the surface and contour plots demonstrating the influence of process variables on transesterification reaction of MNEO using Fe_3_O_4_@CPD–SO_4_, while Fig. 9 represents the heat-map Pearson correlation derived from these plots. An overview of Fig. 8 reveals that biodiesel yield was at least >75%, underscoring the significant influence of each process parameter in present system. According to Fig. 8(a) and (e), biodiesel yield progressively increased from 35% at lower limit of catalyst wt (1 wt%) up to a partial-equilibrium stage of approximately 75% at 4.0 wt% of Fe_3_O_4_@CPD–SO_4_ catalyst. Beyond this amount of catalyst, there was no significant improvement in the yield of biodiesel until termination of the process at the upper limit of 5 wt%. The iso-positive effect recorded throughout the range of catalyst weight corresponded with the positive numerical correlation (0.1950) given in heat-map correlation (Fig. 9) between catalyst weight and biodiesel yield. The increase in biodiesel yield (Fig. 8(a) and (e)) resulting from catalyst weight could be attributed to availability of more active sites which obviously facilitated the conversion of triglycerides of MNEO into biodiesel.^9^ The reduced conversion rate (Fig. 8(a) and (e)) is due to increased viscosity of the reaction liquor resulting from incremental addition of catalyst. This increase viscosity hinders mass transfer between reacting species leading to reduced yield of biodiesel.^1^

The influence of reaction temperature was investigated from 50 °C to 90 °C as demonstrated in combination with other process factors in Fig. 8(a) and (e). An overview of these results highlights the quadratic influence temperature exerted with respect to biodiesel yield. Accordingly, in Fig. 8(c) and (g), biodiesel yield reached 85.8% following a gradual increase in temperature from 50 °C to 63.8 °C. Beyond 63.8 °C, biodiesel yield declined significantly to a value of < 60% at 90 °C reaction temperature. It has been documented that increase in temperature increases the rate of reaction as postulated by Arrhenius equation. In addition to this, increase in temperature decreases the viscosity of esterified oil feedstock, facilitating the mobility of both methanol and MNEO molecules which results in enhanced yield of biodiesel.^17^ Decrease in biodiesel output could be as a result of partial vaporization of methanol which climaxed around its boiling point, leading to a decreased amount of liquid phase methanol available for methanolysis. According to Elgharbawy *et al.*,^95^ methanol fosters the contact between triglyceride molecules and methanol. The effectiveness of this contact obviously reduced with the reduction of methanol volume in liquid phase at temperature beyond 63.8 °C. It is interesting to note that the bubble point of methanol (63.8 °C) is significantly close to the optimum temperature obtained here. The notable quadratic effect corresponded accordingly with a non iso-positive effect of −0.1920 correlating the impact of temperature on biodiesel in Fig. 9.

Fig. 8(b)–(d) and (f)–(h), demonstrates the combined influence of time and cat. wt., temp. and molar ratio, respectively, on the methanolysis of MNEO. Also, according to the information in the heat-map Pearson correlation data (Fig. 9), temperature has a slightly negative impact (−0.0976) on the yield of biodiesel. This negative impact corresponds to a graphical quadratic effect on the conversion of triglycerides to biodiesel. Accordingly, at a molar ratio of 30 : 1, biodiesel yield increased from 91% (at 0.5 h) to a peak of 96.03% (at 1.87 h). Even with more increase in reaction time, biodiesel yield significantly declined to value of 64.14% at a reaction time of 4.0 h. From the basic knowledge of chemistry, increase in reaction time enhances the mixing and dispersion of reacting species, allowing for mass transfer between methanol and MNEO molecules. This phenomenon explains the increased yield in biodiesel relative to progressive increase in reaction time. The decline in the yield of methanolysis product could be due to the consolidated negative effects of loss of esters from product to reaction stream and blockage of Fe_3_O_4_@CPD–SO_4_ catalyst pores by glycerin molecules, leading to a shift in reaction equilibrium to the left hand side.^96^

The methanol- to-oil molar ratio exhibited the highest (0.8615) iso-positive effect on the conversion of MNEO triglycerides to biodiesel. This implies that throughout the reaction levels of molar ratio, biodiesel yield continued to increase from 36% (at 6 : 1) up to 95.54% (at 30 : 1). Given that the transesterification process for producing biodiesel is reversible altering the state of equilibrium by adding sufficient methanol into the reactants might boost the yield of the final product. Improved output of biodiesel is found when the quantity of methanol is increased because the reverse reaction becomes less preferred over the forward step, based on Le Chatelier's principle.^97^

#### 3.3.5. Feature evaluation using SHAP

**Fig. 10:**
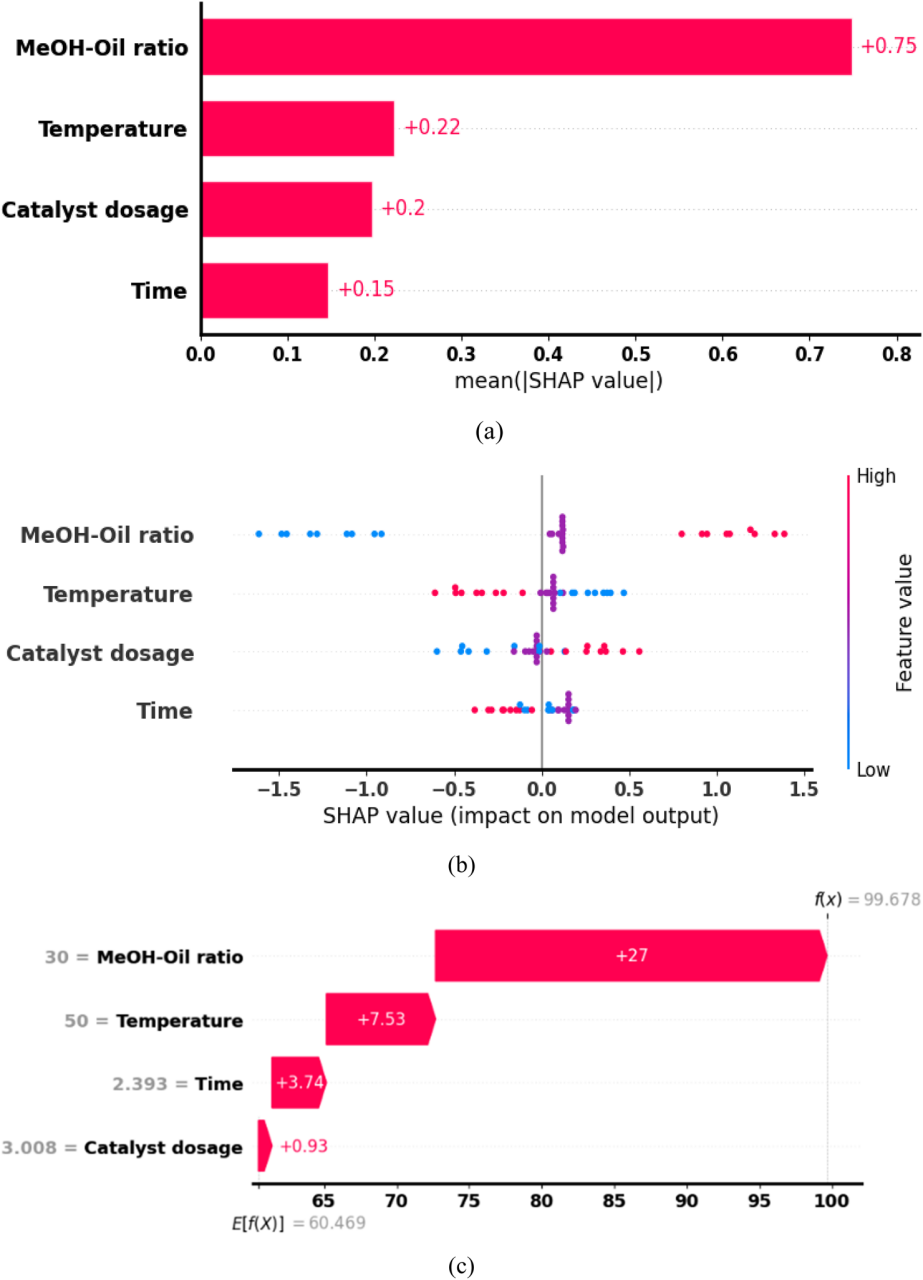
SHAP results for (a) feature ranking plot, (b) beeswarm plot, (c) waterfall plots.

SHAP analysis employs feature ranking and Beeswarm plots to portray the importance and influence, respectively, of each process variable on predicted yield of biodiesel. The feature ranking plot in Fig. 10(a) illustrates that molar ratio is the most important factor in MNEO transesterification reaction. This is likely due to the crucial role of methanol reactant in providing the alkyl group needed in ester formation. Similar observation have been reported by Betiku *et al.*^98^ Following molar ratio is reaction temperature and catalyst weight (catalyst dosage). The importance of these two factors is anchored on their unique roles in transesterification reaction. For instance, the importance of temperature is anchored on its ability to enhance mass transfer during transesterification, while catalyst speed up the rates of this mass transfer operation. The least important process factor according to the feature ranking in Fig. 10(a) is reaction time. Similar observations have been reported by Sambasivam *et al.*,^99^ where it was noted that transesterification reaction time was the least important factor. It should be noted that reaction time solely influences the rate at which the process approaches equilibrium, rather than the total yield after equilibrium is reached, making it less significant in the development biodiesel compared to other variables.

According to the influence (impact) ranking of each feature demonstrated in the Beeswarm plot of Fig. 10(b), methanol to oil molar ratio maintained the lead as the most impactful feature in transesterification of MNEO. According to Guo *et al.*,^63^ blue marks indicate low value impact of each input attributes, whereas red marks indicate high value impact. Consequently, a closer comparison of Fig. 10(a) and (b) reveals that although reaction temperature is more important than catalyst dosage, its influence was slightly greater than that of reaction temperature on the prediction outcome of biodiesel yield. It is noteworthy that the impact of reaction time was the least, corresponding to the feature ranking in Fig. 10(a). Specifically, the beeswarm result implies that positive SHAP values of biodiesel yield were favoured by high levels of molar ratio, while negative SHAP values were associated with low amounts of molar ratio. This observation corroborated with the discussions in Section 3.3.4., further consolidated the plausibility that increasing the volume of methanol actually facilitated equilibrium displacement by favoring the reaction, resulting in increased yield of biodiesel.^97^ Furthermore, Fig. 10(b) reveal that high SHAP values were obtainable at low temperatures, as opposed to high temperature values. This phenomenon further supports earlier observation that increasing this feature, in effect, led to the reduction of the top-ranking feature (molar ratio) within the reaction vessel. This reduction in molar ratio was triggered by volatilisation of methanol at elevated temperatures, causing a reduction in liquid phase methanol available for transesterification reaction. For catalyst weight feature, the beeswarm findings indicated that if improved yield of biodiesel is desired, higher values of catalyst weight will be required. The mechanistic implication here is that the availability of more active catalytic sites obviously favoured the accelerated conversion of MNEO to biodiesel.^9^Fig. 10(c) depicts the SHAP waterfall plot. This figure exemplifies the contribution of each feature in predicting optimum yield of biodiesel. Accordingly, feature contribution to predicted optimum followed the order molar ratio > temperature > time > catalyst dosage. This implies that methanol-to-oil molar ratio was the most influential process feature in MFRO algorithm by a magnitude of +27, while catalyst weight having a magnitude of +0.93 was the least.

### 3.4. Biodiesel characterization

The physicochemical properties of MNEO methyl ester synthesized at optimum conditions was characterized and compared to compared to commercial diesel and ASTM D-6751 standards (Table 10). Distinct fuel properties including viscosity, cloud point, and pour point were found to be within the permissible range to guarantee unhindered flow at low temperature. Furthermore, an acid value of 0.33 mg KOH per g oil not only demonstrates a significant reduction from 9.018 mg KOH per g oil, it also gives credence to the fact that there will be no corrosion associated with the combustion of MNEO methyl ester. The level of moisture content (0.023%) and saponification value indicates a good quality fuel with easy combustion characteristics. An indication of fuel ignition quality and quantity of heat energy associated with its combustion was given by cetane number and higher heating value, respectively. From these values, MNEO methyl ester possess a satisfactory ignition property and acceptable heat energy associated with the combustion dynamics. These properties highlight the potentials of MNEO methyl ester as a suitable liquid fuel for internal combustion engines.

**Table 10:** Physicochemical analysis of MNEO methyl ester

Property	MNEO	Biodiesel	Comercial diésel	ASTM D6751
Acid value (mg KOH per g oil)	9.018 ± 0.01	0.33 ± 0.00	—	<0.5
FFA (%)	4.404 ± 0.021	0.17 ± 0.01	—	<0.25
Iodine value (g I_2_/100 g oil)	68.533 ± 0.01	52.24 ± 0.01	—	—
Cloud point (°C)	—	7 ± 0.00	Max + 5	−3 to 12
Pour point (°C)	—	4 ± 0.00	—	−15 to 10
Saponification value	184.455 ± 0.02	127.45 ± 0.03	—	—
Density @ 28 °C (g cm^−3^)	0.9031 ± 0.02	0.873 ± 0.01	0.8300–0.8601	—
Viscosity (mm^2^ s^−1^)	—	4.41 ± 0.01	3.15 ± 0.03	3.5 to 5.0
Moisture content (%)	—	0.023 ± 0.01	—	<0.03
Cetane number	—	53.9 ± 0.01	46–55	>47
Higher heating value (MJ kg^−1^)	—	38.95 ± 0.01	44.0–45.4	>35
Methyl ester content (%)	—	98.87	—	>96.5

### 3.5. Catalyst reusability and characterization

Two essential qualities of catalysts for manufacturing purposes are their capacity for reuse and catalytic efficiency. TheFe_3_O_4_@CPD–SO_4_ re-usability investigation was carried out in the optimum parameters specified by MRFO. The MNEO biodiesel output values from five successive cycles regardless of any further modification are displayed in Fig. S1. The gradual saturation of active spots on Fe_3_O_4_@CPD–SO_4_ catalyst surface by transesterification intermediates may be responsible for the apparent little drop in biodiesel output following each cycle.^17^ Activation reagents (sulfur ions) eventually seep towards the reaction liquor as a result of this obstruction, decreasing the Fe_3_O_4_@CPD–SO_4_ catalytic performance. A generic overview of Fig. S5 highlights a satisfactory performance of Fe_3_O_4_@CPD–SO_4_ catalyst. This satisfactory performance was authenticated by a negligible 15.77% reduction in biodiesel yield after 4 cycles of non-regenerative reaction. However, at the end of 7th cycle, biodiesel yield of 62.33% was recorded, showing a significant decline in Fe_3_O_4_@CPD–SO_4_ catalytic activity.

**Fig. 11:**
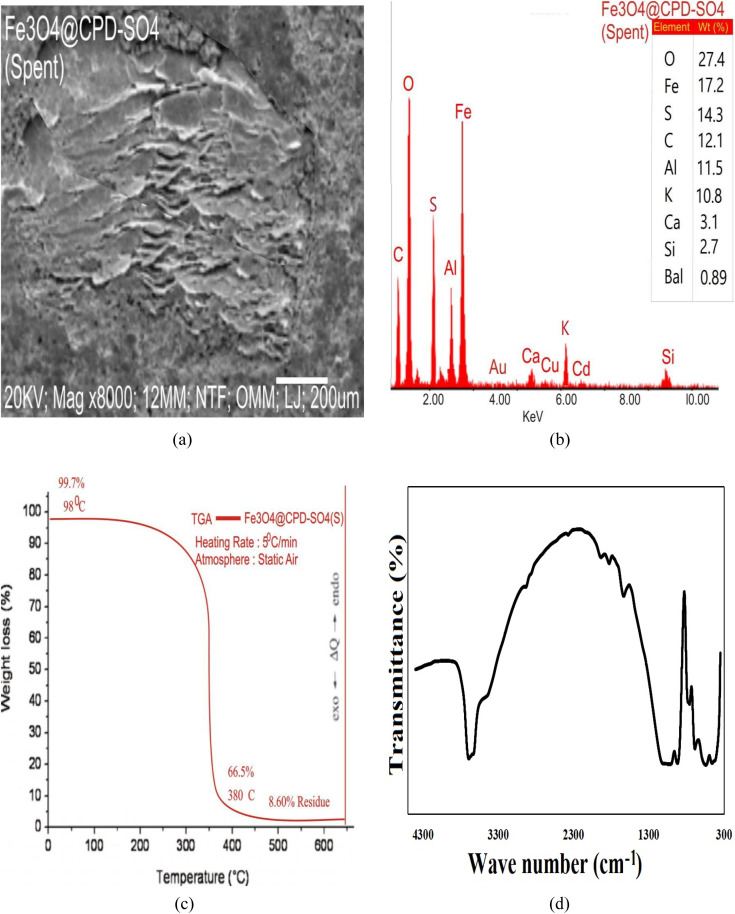
Instrumental analysis of spent-Fe_3_O_4_@CPD–SO_4_ catalyst for (a) SEM, (b) EDX, (c) TGA, (d) FTIR.

Characterization of spent Fe_3_O_4_@CPD–SO_4_ catalyst and biodiesel product are presented in Fig. 11, and Table 7, respectively. The SEM image depicts a swollen ridge-like matrix illustrating imbibition of reacting liquor. Furthermore, Fig. 11(a) portrays the disappearance of porous dark array, demonstrating a probable saturation of catalyst active sites as a consequence of transesterification reaction. The EDX results show discernible reductions in calcium, sulfur, potassium and iron contents compared to Fig. 4(f). These reductions not only demonstrate the active participation of Fe_3_O_4_@CPD–SO_4_ catalyst in methanolysis of MNEO, it further corroborates the postulations concluded in the re-usability study that active elements percolated progressively towards the reaction liquor during the process. Thermogravimetric analysis (Fig. 11(c)) show that spent Fe_3_O_4_@CPD–SO_4_ retained its thermal stability even after fifth cycle of reaction. FTIR spectrum of spent Fe_3_O_4_@CPD–SO_4_ (Fig. 11(d)) corroborate favorably with the findings of EDX. The infrared spectrum shows the disappearance of sulfonated polysaccharides in wave number 805 cm^−1^, while the reduction in sulfonated polymeric aromatic compounds was depicted by the peak at 1250 cm^−1^. These deformations in wavenumber authenticate the deployment of Fe_3_O_4_@CPD–SO_4_ in transesterification of MNEO. Furthermore, the reduced catalytic activity and saturation of active sites was additionally evidenced by the decreased vibrational peak at 546 cm^−1^ (Fe–O) and 1040 cm^−1^ (C–O stretching of polysaccharides).

## 4. Conclusion

The current research presents the results of one-pot transesterification of reaction of optimally formulated MNEO. Optimal formulation was executed using mixture design technique to obtain a blending ratio of 21.31 : 18.45 : 60.24 on v/v basis of WCO : RO-POME : CO, respectively. In addition to the physicochemical properties, three major parameters including acid value, density, and iodine value were used to adjudge the best oil formulation. The preparation of catalyst species possessing bi-functional catalytic potentials was successfully carried out using calcination, sulfonation, and magnetization. Instrumental characterization using FTIR, TGA, SEM-EDX, and XRD authenticated the relevance and effectiveness of each activation process, further giving credence to the potentials of Fe_3_O_4_@CPD–SO_4_ in one step methanolysis of MNEO. Machine learning modeling of the process was effectively done using ANN, SVR and XGB models. Statistical and error appraisal techniques established the marginal superiority of XGB over ANN, and SVR in capturing the nonlinear nature of the system. Feature analysis was done using SHapely additive exPlanations (SHAP). Feature ranking and Beeswarm plots indicated that MR was both the most important and most impactful process parameter. Process optimization using MRFO predicted an optimum yield of 99.68% at a methanol-to-oil ratio of 30.0, temperature of 50 °C, reaction time of 2.4 h, and catalyst dosage of 3.01%. Triplicate validation experiments authenticated the optimum prediction of MFRO at 98.16 ± 0.11%, and physicochemical characterization confirmed the suitability of MNEO methyl ester in internal combustion engines.

## Author contributions

Paschal Enyinnaya Ohale: investigation, writing – original draft, data analysis, writing – reviewing and editing. Andrew Nosakhare Amenaghawon: conceptualization, methodology, writing – original draft, reviewing and editing, supervision. Thomas Okpo Kimble Audu: conceptualization, methodology, reviewing and editing, supervision. Favour Ugbodu: data analysis. Lilian Chikasi Okonkwo investigation, validation. Oghenerukevwe Jeffrey Oghenehwosa: writing – reviewing and editing.

## Conflicts of interest

The authors declare that they have no conflict of interest.

## Supplementary Material

RA-015-D5RA07881D-s001

## Data Availability

All data generated or analyzed during this study are included in this published article or the supplementary information (SI). Supplementary information: tables accompanying our mansucript including mixture design matrix, ML modeling data, hyperparameter data, comparative transesterification of mixed nonedible oil feedstock, and catalyst reusability plot. See DOI: https://doi.org/10.1039/d5ra07881d.
