# Supplementary material for: Machine learning-informed one-pot biodiesel synthesis from an optimally formulated mixed non-edible oil feedstock over magnetic sulfonated biobased catalyst
Source: RSC Adv. 2025 Dec 18;15(59):50856–80. doi: 10.1039/d5ra07881d (PMC12712571; doi:10.1039/d5ra07881d)
Supplement: RA-015-D5RA07881D-s001 [file RA-015-D5RA07881D-s001.pdf]

Supplementary Information (SI) for RSC Advances.
This journal is © The Royal Society of Chemistry 2025

Machine learning-informed one-pot biodiesel synthesis from an optimally formulated mixed non-edible oil feedstock over magnetic sulfonated biobased catalyst

*[1,2,3]Paschal Enyinnaya Ohale, **[1]Andrew Nosakhare Amenaghawon, [1]Thomas Okpo Kimble Audu, [1]Favour Ugbodu, [2,3]Lilian Okonkwo and [1]Oghenerukevwe Jeffrey Oghenehwosa

[1]Bioresources Valorization Laboratory, Department of Chemical Engineering, Faculty of Engineering, University of Benin, Benin city, Edo state, Nigeria.

[b]Department of Chemical Engineering, Faculty of Engineering, Nnamdi Azikiwe University, PMB 5025, Awka, Nigeria.

[c]PECO Research Investigators Sci-lab, Anambra State, Nigeria.

*pe.ohale@unizik.edu.ng

**andrew.amenaghawon@uniben.edu

ORCID: P.E Ohale: https://orcid.org/0000-0001-5332-7742

ORCID: A. N. Amenaghawon: https://orcid.org/0000-0003-0433-0114

Table S1: Mixture design matrix for formulation of oil blend

Run	Oil ratios (%)			Responses							
	WCO	RO-POME	CO	FFA (%)		Acid value (mg KOH/g)		Density (g/cm^3)		Iodine Value (g/100g)	
				Exp.	Pred.	Exp.	Pred.	Exp.	Pred.	Exp.	Pred.
1	0.00	50.00	50.00	8.22	8.00	16.38	16.68	0.8743	0.8807	74.96	74.50
2	50.00	50.00	0.00	10.1	9.48	16.66	16.49	0.8687	0.8714	52.29	51.10
3	16.67	16.67	66.67	4.32	4.08	8.61	8.51	0.8932	0.8907	79.55	80.53
4	66.67	16.67	16.67	4.88	5.13	9.72	9.68	0.8786	0.8790	62.71	63.04
5	30.00	45.00	25.00	8.2	8.17	16.66	15.63	0.8767	0.8758	65.25	65.46
6	0.00	0.00	100.00	1.81	2.03	3.61	3.68	0.9653	0.9653	80.92	80.95
7	33.33	33.33	33.33	6.41	6.57	12.77	12.78	0.8808	0.8794	70.00	70.58
8	50.00	0.00	50.00	3.35	3.15	8.05	7.17	0.8907	0.8879	73.83	72.72
9	45.00	25.00	30.00	5.16	5.75	10.27	11.12	0.8812	0.8800	68.74	69.41
10	16.67	66.67	16.67	10.87	11.41	21.65	22.14	0.8712	0.8703	54.71	54.75
11	0.00	100.00	0.00	17.73	17.69	35.26	35.25	0.8613	0.8606	24.84	25.23
12	25.00	30.00	45.00	6.01	5.91	11.38	11.86	0.8848	0.8826	74.32	75.13
13	50.00	0.00	50.00	3.34	3.15	6.66	7.17	0.8817	0.8879	73.25	72.72
14	0.00	50.00	50.00	8.12	8.00	17.21	16.68	0.8833	0.8807	75.53	74.50
15	100.00	0.00	0.00	4.04	3.70	8.05	7.37	0.884	0.8795	51.83	51.86
16	100.00	0.00	0.00	3.34	3.70	6.66	7.37	0.8761	0.8795	51.63	51.86

Table S2: with Inputs, Experimental output and predicted output

Inputs				Exp output (%)	Predictions (%)		
Temperature (°C)	Catalyst dosage (wt.%)	MeOH-oil ratio	Time	Biodiesel yield	ANN	SVR	XGB
60.0	2.0	12.0	1.38	47.0264 tr	48.6064	46.4977	48.3362
80.0	2.0	12.0	1.38	43.5159 tr	43.7270	42.9872	39.7983
60.0	4.0	12.0	1.38	33.2807 tr	35.6220	40.0395	32.6124
80.0	4.0	12.0	1.38	40.5441 tr	42.1045	41.0715	38.1345
60.0	2.0	24.0	1.38	84.0970 ts	84.3588	82.8261	83.5836
80.0	2.0	24.0	1.38	69.8490 tr	70.8652	70.3782	63.5403
60.0	4.0	24.0	1.38	91.5153 tr	91.4740	90.9855	93.5690
80.0	4.0	24.0	1.38	88.0412 ts	86.7229	85.3623	87.4041
60.0	2.0	12.0	3.13	42.5897 tr	42.8704	41.5399	43.6660
80.0	2.0	12.0	3.13	28.7631 tr	35.5562	34.1055	27.4816
60.0	4.0	12.0	3.13	39.9434 tr	39.8967	40.4688	37.0202
80.0	4.0	12.0	3.13	36.8909 tr	38.0762	37.4225	35.3868
60.0	2.0	24.0	3.13	76.5200 tr	79.4635	75.9929	73.4097
80.0	2.0	24.0	3.13	41.9800 ts	46.2474	58.0803	44.8880
60.0	4.0	24.0	3.13	88.6400 tr	88.4517	88.1206	92.3299
80.0	4.0	24.0	3.13	80.6600 ts	82.9021	76.7634	78.1776
50.0	3.0	18.0	2.25	63.7107 tr	64.8033	64.2361	72.6766
90.0	3.0	18.0	2.25	49.2900 tr	45.5234	52.5106	53.2205
70.0	1.0	18.0	2.25	53.1200 tr	54.5907	54.0869	55.7162
70.0	5.0	18.0	2.25	72.4300 tr	72.5321	71.3797	74.2196
70.0	3.0	6.0	2.25	20.5900 tr	25.6826	21.1241	26.3954
70.0	3.0	30.0	2.25	95.3500 tr	94.2742	95.8685	94.9045
70.0	3.0	18.0	0.50	53.8700 tr	56.6208	62.0966	57.9184
70.0	3.0	18.0	4.00	58.8740 tr	44.6907	53.7117	59.4104
70.0	3.0	18.0	2.25	66.5800 ts	67.4591	66.7569	66.7125
70.0	3.0	18.0	2.25	62.5590 tr	67.4591	66.7569	66.7125
70.0	3.0	18.0	2.25	70.8000 tr	67.4591	66.7569	66.7125
70.0	3.0	18.0	2.25	77.2000 ts	67.4591	66.7569	66.7125
70.0	3.0	18.0	2.25	67.2900 tr	67.4591	66.7569	66.7125
70.0	3.0	18.0	2.25	67.7130 tr	67.4591	66.7569	66.7125

tr = Training data
ts = testing data

Table S3: Table of optimized hyperparameters

Model	Hyperparameters	Final optimized value	Total execution time (s)
	Kernel_initializer	normal	
	model optimizer	RMSprop	
	epochs	300	
	Convergence epoch	5	
	MSE of best validation performance	0.0404841	
	layer 1 number of neurons	56	
ANN	layer 1 activation function	relu	2595.2
	layer 2 number of neurons	80	
	layer 2 activation function	relu	
	layer 3 number of neurons	32	
	layer 3 activation function	relu	
	output layer number of neurons	1	
	output activation function	relu	
	C	1.06347	
SVR	ϒ	0.096263556191	238
	Kernel	rbf	
	random state	42	
	max depth	10	
	Sub-sample	0.82	
	min_child_weight	3.0	
XGB	Reg_α	0.06746	154.7
	Reg_λ	0.04349	
	ϒ	0.000365222	
	n estimators	1400	
	random state	84	

Data and code repository

https://github.com/ucbabe/Pasc-Data-Biodiesel-Modelling-Results/tree/main

Table S4: Comparison of heterogeneous catalysis of mixed oil feedstock from recent publications and current work

Mixed oil Feedstock	Catalyst source	Reaction temperature (°C)	Catalyst concentration (wt%)	Reaction time (min)	Methanol-to-oil ratio	Biodiesel yield (%)	Reference
Waste cooking oil (21.31%)<br>Castor oil (18.45%)<br>Recovered oil from palm oil mill effluent (60.24%)	Poultry droppings	55	3.35	147.6	26.83:1	98.16	Prresent study
Jatropha curcas oil (50%)<br>Pongamia pinnata oil (50%)	Banana waste	65	9	10 ± 2	9:1	96.34 ± 1.30	(Basumatary *et al.*, 2025)
Pig fat oil (70%)<br>Tallow seed oil (30%)	Waste green coconut husks -AC	80	2	70	5.76:1	97.67	(Babatunde *et al.*, 2025)
Jatropha (20%)<br>Karanja (20%)<br>Waste cooking oil (60%)	KOH	70	1	120	8:1	93	(Husaini *et al.*, 2025)
Castor oil (80%)<br>Cotton seed (20%)	Dolomite	60	-	240	6:1	87.7	(Vieira *et al.*, 2025)
Soybean oil (20%)<br>Sun flower oil (20%)<br>Canola oil (20%)<br>Pongamia oil (20%)<br>Jatropha oil (20%)	Waste black gram plant	65	10	96 ± 8.4	9:1	94.79 ± 0 27	(Brahma *et al.*, 2024)
Waste cooking oil (50%)<br>Baobad oil (50%)	Waste Avocado Peels	60	2.73	51	14.5:1	92.85	(Etim & Musonge, 2024)
Soybean (33.33)<br>Jatropha (33.33)<br>Pongamia (33.33)	Areca nut leaf $ash - K_2CO_3$	65	15	201±9	9:1	96.57 ± 0.81	(Boro *et al.*, 2024)

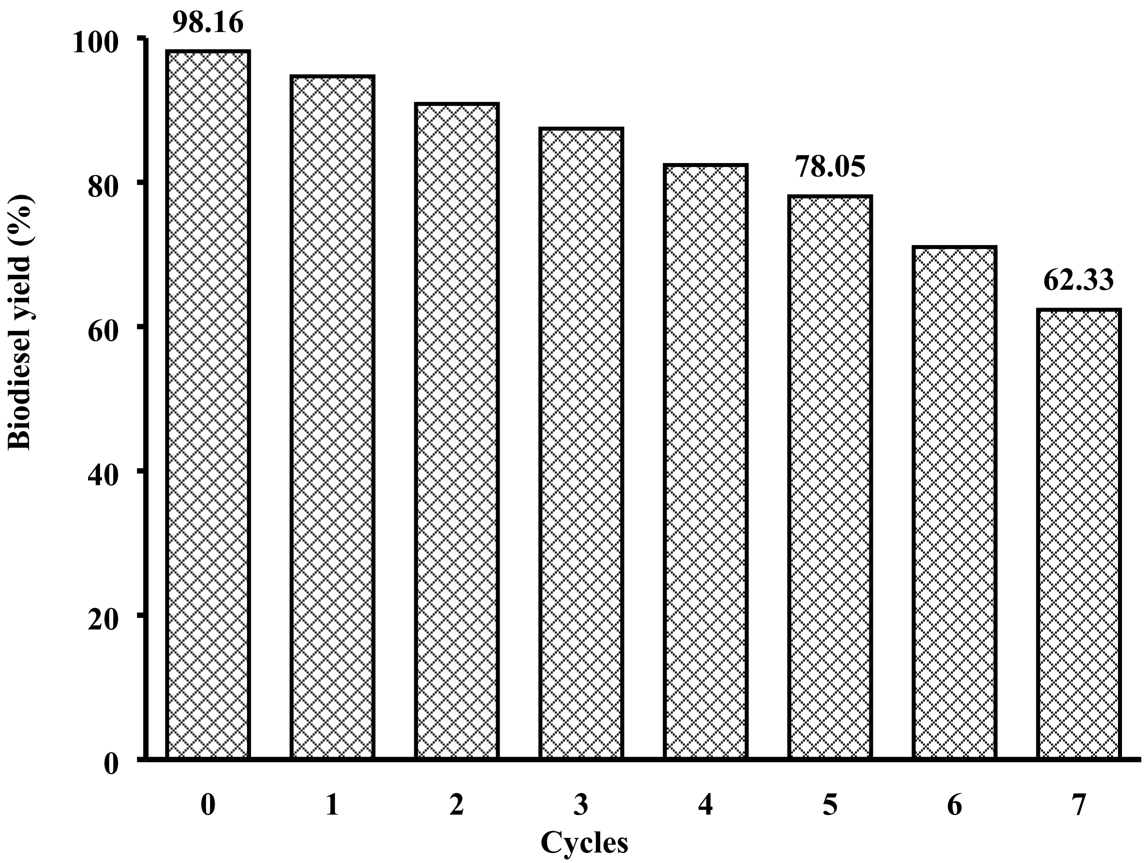

Figure S1: Catalyst reusability studies

References

Babatunde, E., Enomah, S., Akwenuke, O., Adepoju, T., Okwelum, C., Mundu, M., Aiki, A., & Oghenejabor, O. (2025). Novel-activated carbon from waste green coconut husks for the synthesis of biodiesel from pig fat oil blends with tallow seed oil. *Case Studies in Chemical and Environmental Engineering, 11*, 101058.

Basumatary, S. F., Das, B., Brahma, S., & Basumatary, S. (2025). Musa ABB (Kachkal) banana waste derived heterogeneous nanocatalyst for transesterification of binary oil mixture of Jatropha curcas and Pongamia pinnata to biodiesel. *Bioresource Technology Reports, 29*, 102018.

Boro, S., Das, B., Brahma, S., Basumatary, B., Basumatary, S. F., & Basumatary, S. (2024). Biodiesel production using areca nut (Areca catechu L.) leaf ash-K2CO3 catalyst via transesterification from an oil blend of three different feedstocks. *Sustainable Chemistry for the Environment, 8*, 100164.

Brahma, S., Basumatary, B., Mushahary, B. C., Basumatary, S. F., Das, B., Selvaraj, M., & Basumatary, S. (2024). Vigna mungo (L.) hepper as heterogeneous catalyst for generation of biodiesel from a mixture of multiple oil feedstocks. *International Journal of Energy Research, 2024*(1), 7407501.

Etim, A. O., & Musonge, P. (2024). Synthesis of a highly efficient mesoporous green catalyst from waste Avocado peels for biodiesel production from used cooking–baobab hybrid oil. *Catalysts, 14*(4), 261.

Husaini, S., Kadire, A., Verma, R. K., & Pydimalla, M. (2025). Biodiesel production from non-edible mixed oils: a sustainable approach using jatropha, karanja and waste cooking oil. *Bulletin of Materials Science, 48*(2), 58.

Vieira, R. B., Gomes, F. d. S., Xavier, R. L. L., Lima, V. H. S., Farias, A. C. P., Barros, J. F. d. S., Valentini, A., & Rodrigues dos Santos, R. C. (2025). Oxidative Stability and Molecular Reactivity of Biodiesel from Castor and Cottonseed Oil Blends by Homogeneous and Heterogeneous Catalysis. *Energy Technology*, 2500287.
